# Consecutive Inhibition of ISG15 Expression and ISGylation by Cytomegalovirus Regulators

**DOI:** 10.1371/journal.ppat.1005850

**Published:** 2016-08-26

**Authors:** Ye Ji Kim, Eui Tae Kim, Young-Eui Kim, Myoung Kyu Lee, Ki Mun Kwon, Keun Il Kim, Thomas Stamminger, Jin-Hyun Ahn

**Affiliations:** 1 Department of Molecular Cell Biology, Samsung Biomedical Research Institute, Sungkyunkwan University School of Medicine, Suwon, Republic of Korea; 2 Department of Biological Sciences, Sookmyung Women's University, Seoul, Republic of Korea; 3 Institute for Clinical and Molecular Virology, University of Erlangen-Nuremberg, Schlossgarten, Erlangen, Germany; Washington University School of Medicine, UNITED STATES

## Abstract

Interferon-stimulated gene 15 (ISG15) encodes an ubiquitin-like protein that covalently conjugates protein. Protein modification by ISG15 (ISGylation) is known to inhibit the replication of many viruses. However, studies on the viral targets and viral strategies to regulate ISGylation-mediated antiviral responses are limited. In this study, we show that human cytomegalovirus (HCMV) replication is inhibited by ISGylation, but the virus has evolved multiple countermeasures. HCMV-induced ISG15 expression was mitigated by IE1, a viral inhibitor of interferon signaling, however, ISGylation was still strongly upregulated during virus infection. RNA interference of UBE1L (E1), UbcH8 (E2), Herc5 (E3), and UBP43 (ISG15 protease) revealed that ISGylation inhibits HCMV growth by downregulating viral gene expression and virion release in a manner that is more prominent at low multiplicity of infection. A viral regulator pUL26 was found to interact with ISG15, UBE1L, and Herc5, and be ISGylated. ISGylation of pUL26 regulated its stability and inhibited its activities to suppress NF-κB signaling and complement the growth of UL26-null mutant virus. Moreover, pUL26 reciprocally suppressed virus-induced ISGylation independent of its own ISGylation. Consistently, ISGylation was more pronounced in infections with the UL26-deleted mutant virus, whose growth was more sensitive to IFNβ treatment than that of the wild-type virus. Therefore, pUL26 is a viral ISG15 target that also counteracts ISGylation. Our results demonstrate that ISGylation inhibits HCMV growth at multiple steps and that HCMV has evolved countermeasures to suppress ISG15 transcription and protein ISGylation, highlighting the importance of the interplay between virus and ISGylation in productive viral infection.

## Author Summary

Type I IFN response is a front-line defense against virus infection. Activation of type I IFN signaling leads to expression of a subset of cellular proteins encoded by interferon-stimulated genes (ISGs). ISG15 encodes an ubiquitin-like protein that is covalently conjugated to protein lysine residues. ISG15 modification (ISGylation) of a protein causes changes of protein function. ISGylation is known to inhibit the replication of many viruses, although pro-viral effects of ISGylation are also reported. Given that ISG15 and the enzymes involved in ISGylation are strongly induced upon virus infection, understanding the interplay between virus and ISGylation is an important issue in virus-host interaction. Nevertheless, viral substrates of ISG15 and viral strategies to regulate ISGylation-mediated antiviral responses are limited to only a few examples. In this study we demonstrate that ISGylation suppresses human cytomegalovirus (HCMV) infection but the virus is armed with countermeasures that consecutively reduce ISG15 transcription and protein ISGylation. Interestingly, a viral ISG15 target is found to inhibit ISGylation. This study highlights that ISGylation is a critical innate immune response against HCMV infection and interfering with ISG15-mediated anti-viral immunity is critical for productive viral infection.

## Introduction

Type I interferons (IFNs) are multifunctional cytokines that represent crucial components of the innate immune response to viral infection. Recognition of viral infection by host cells induces the synthesis of type I IFNs. Secreted IFNs interact with IFN receptors on target cells, triggering a signaling cascade that involves Janus kinase (JAK) and signal transducer and activator of transcription (STAT) families. Activated STAT1 and STAT2 heterodimerize and bind to IFN regulatory factor 9 (IRF9) to form a complex called IFN-stimulated gene factor 3 (ISGF3). This complex translocates into the nucleus and induces ISGs with diverse antiviral activities by binding to IFN-stimulated response elements (ISREs) in their promoters (for review [1]).

ISG15 was identified as an IFN-inducible ubiquitin homolog. Like ubiquitin, its carboxy-terminal LRLRGG motif is required both for recognition by processing enzymes and covalent conjugation to lysine residues of target proteins. ISG15 modification (also termed ISGylation) is an IFN-stimulated and -regulated process that is found only in higher vertebrate animals and appears to modulate the function of target proteins (for review [2]). UBE1L is the E1 activating enzyme for ISG15 [3], and UbcH8, an ubiquitin E2 conjugating enzyme, also acts as the ISG15 E2 conjugating enzyme [4, 5]. HERC domain and RCC1-like domain containing protein 5 (Herc5), estrogen-responsive finger protein (EFP), and human homolog of *Drosophila ariadne* (HHARI) have been identified as E3 ligases for ISGylation in human cells [6–9]. ISG15, UBE1L, UbcH8, and Herc5 are IFN-inducible [4, 6, 10, 11]. Conjugated ISG15s are removed by an ISG15-specific protease, UBP43 (also known as USP18) [12]. Interestingly, UBP43 is also IFN-inducible [13, 14] and acts as a negative regulator of innate immune responses independent of its protease activity but dependent on its direct interaction with IFNAR2, a subunit of the type I IFN receptor [15].

Antiviral responses involving protein ISGylation have been reported against diverse viruses. Several cellular proteins involved in antiviral signaling, including RIG-I, MDA-5, STAT1, JAK1, IRF3, PKR, Mx1, and RNase L, were also identified or suggested as substrates for ISGylation [16–21]. ISGylation suppressed replication of diverse viruses, such as influenza virus (type A and B) [22–25], human immune deficiency virus (HIV) [26, 27], hepatitis C virus (HCV) [28–30], Japanese encephalitis virus [31], Sindbis virus [23, 32, 33], Ebola VP40 virus-like particle [34, 35], herpes simplex virus type-1 [23], murine γ-herpesvirus 68 [23], vaccinia virus [36], dengue and West Nile viruses [37], porcine reproductive and respiratory syndrome virus [38], Kaposi’s sarcoma-associated herpesvirus (KSHV) [39], and respiratory syncytial virus [40]. However, the antiviral mechanism of ISGylation against specific viruses is poorly understood. Herc5 associates with polyribosomes, and ISGylation appears to be restricted largely to newly synthesized proteins, suggesting that newly synthesized viral proteins may be primary targets of ISG15 [41]. ISGylation of NS1A in influenza A virus disrupted its association with importin-α, which mediates the nuclear import of NS1A, thus inhibiting viral replication [42]. ISGylation also suppressed the release of retrovirus particles by disrupting the budding process-related protein complex (for review [43]). An ISGylation-independent antiviral effect of ISG15 was also demonstrated in Chikungunya virus infection [44].

Although several studies have suggested a general role for ISG15 as an antiviral molecule, proviral effects of ISGylation have also been reported for certain viruses. In Newcastle disease virus (NDV) infection, ISGylation of RIG-I reduced IFN responses as a negative feedback regulation mechanism [45], whereas ISGylation of IRF3 stabilized IRF3 [21]. In addition, enhanced ISGylation by ISG15 overexpression promoted HCV production, while reduced ISGylation by UBE1L- or ISG15-knockdown inhibited HCV production [46, 47]. Therefore, the effects of global protein ISGylation appear to vary among the different viruses. In addition, ISG15 is secreted to the extracellular space as a free unconjugated form [48, 49]. While secretion of ISG15 in granulocytes is shown to activate T cells and natural killer cells to produce IFNγ in mycobacterial infection [50, 51], the role of secreted ISG15 in viral infection is not clear. Furthermore, a role of free ISG15 as a negative regulator that prevents IFNα/β overamplification and auto-inflammation by sustaining UBP43 levels has been suggested in humans but not in mice [52, 53].

Human cytomegalovirus (HCMV) is an opportunistic pathogen that causes severe disease complications and pathologies in newborns and immunocompromised individuals [54]. During productive infection, HCMV gene expression occurs sequentially in three phases: immediate-early (IE), early, and late. IE proteins and virion-associated tegument proteins play key roles in initiating viral gene expression and modulating host cell functions. HCMV employs several mechanisms to counteract IFN production and subsequent ISG activation. IE2 and pp65 inhibit IFN production [55–57], whereas IE1 suppresses the IFN response by directly binding to STAT2 [58–60] and PML [61, 62]. HCMV infection results in a decrease in the levels of JAK1 and p48, two components of the type I IFN signaling pathway [63, 64]. Modulation of the stability and phosphorylation of STAT proteins during HCMV infection was also reported [65, 66].

ISG15 transcription is induced in HCMV infection; however, its regulation during infection, the role of ISGylation in viral growth, and viral targets of ISG15 have not been characterized. In this study, we show that ISG15 expression and ISGylation are initially induced after HCMV infection but later suppressed by viral responses, and that IE1, a viral inhibitor of STAT signaling, plays an important role in reducing ISG15 transcription. By silencing the expression of E1, E2, and E3 ISGylation enzymes and of an ISG15 protease, we also demonstrate that ISGylation inhibits HCMV growth at multiple steps, including viral gene expression and virion release. Furthermore, we show that pUL26, a viral tegument protein, interacts with ISG15, E1, and E3 and is modified by ISG15, which inhibits pUL26 activity to promote viral growth. Moreover, we reveal that expression of pUL26 is able to suppress ISGylation induced by virus infection. Our results indicate that HCMV has evolved countermeasures to suppress ISG15 transcription and protein ISGylation, highlighting the important of the interplay between virus and ISG15 signaling during virus infection.

## Results

### Time course of ISG15 expression and protein ISGylation during HCMV infection

**Fig 1:**
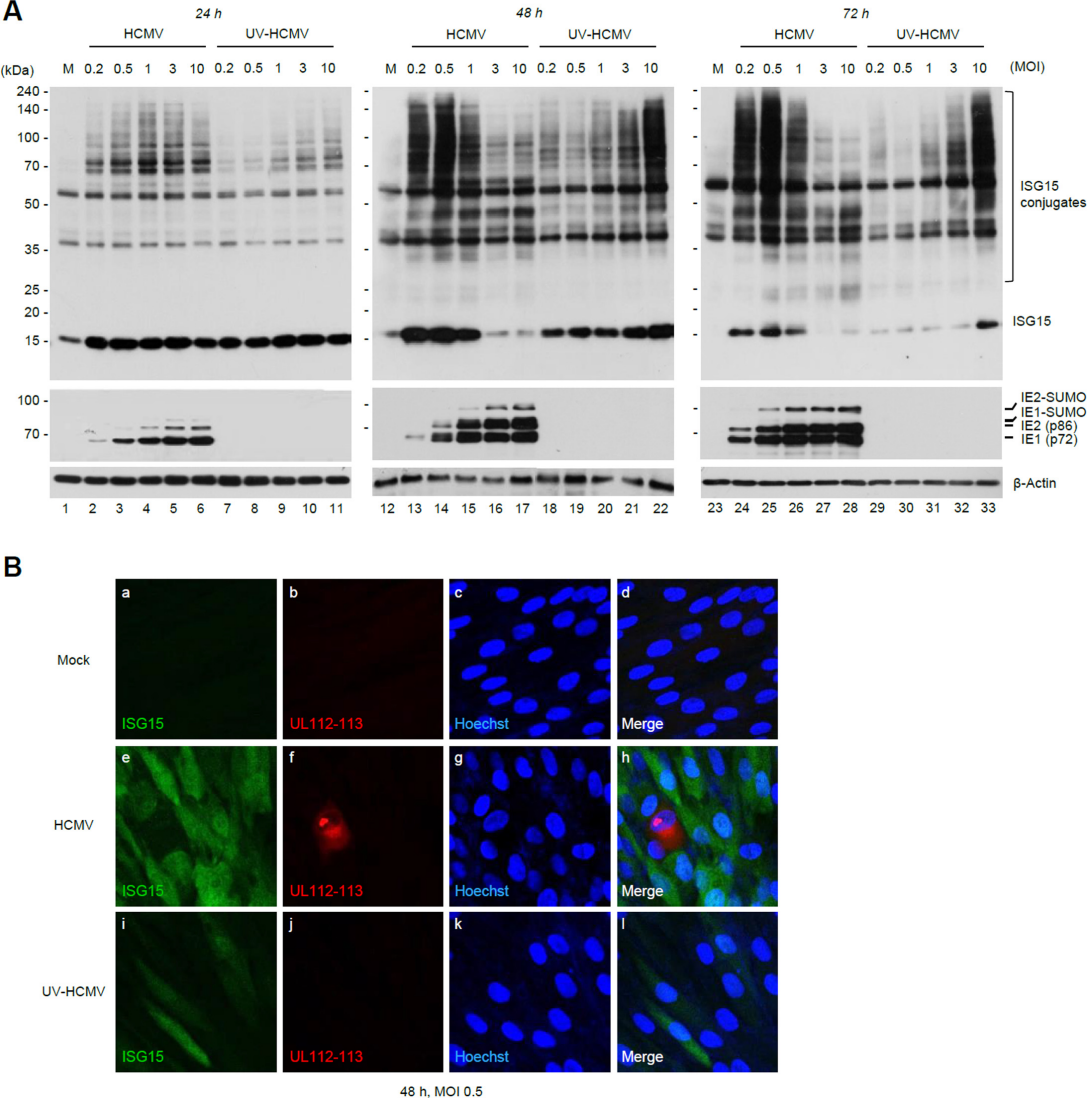
Time course of ISG15 expression and protein ISGylation during HCMV infection. (A) HF cells were mock-infected (M) or infected with HCMV (Towne) or UV-HCMV at different MOIs (0.2 to 10) as indicated. Cell lysates were prepared at 24, 48, and 72 h after infection and immunoblotted with antibodies for ISG15, IE1/IE2, or β-actin (a loading control). (B) HF cells were mock-infected or infected with HCMV or UV-HCMV at an MOI of 0.5 for 48 h. Cells were fixed with methanol and double-label IFA was performed with antibodies for ISG15 and UL112-113. Hoechst stain was used to stain cell nuclei. The images were obtained by confocal microscopy.

The time course of ISG15 expression and protein ISGylation during HCMV infection were investigated with different multiplicity of infections (MOIs). In human fibroblast (HF) cells infected with HCMV (Towne), the levels of ISG15 and protein ISGylation were elevated by 24 h at all MOIs tested (MOIs of 0.2 to 10) (Fig 1A, lanes 2–6). At 48 and 72 h after infection, even greater levels of ISG15 expression and protein ISGylation were observed at relatively low MOIs (0.2, 0.5, and 1) (Fig 1A, lanes 13–15 and 24–26); however, the levels of free ISG15 and ISG15 conjugates at high MOIs (3 and 10) were much lower than those at low MOIs (Fig 1A, lanes 16–17 and 27–28). The time course of ISG15 expression and protein ISGylation was also examined in cells infected with UV-inactivated virus (UV-HCMV). In UV-HCMV infection, the levels of ISG15 and ISG15 conjugates were elevated at 24 and 48 h and correlated proportionally with MOI (Fig 1A, lanes 7–11 and 18–22). Levels of free ISG15 at 72 h induced by UV-HCMV were lower than those at 48 h, probably due to the termination of signaling (Fig 1A, compare lanes 29–33 and 18–22). The lack of viral gene expression in UV-HCMV infection was verified by the absence of viral IE protein expression. Collectively, these results comparing HCMV and UV-HCMV infection demonstrate that ISG15 expression and protein ISGylation are initially induced by HCMV infection, but are subsequently suppressed in a manner dependent on viral gene expression.

Notably, we observed a greater induction of ISG15 and protein ISGylation with HCMV than with UV-HCMV at low MOIs (MOIs of 0.2, 0.5, and 1) (Fig 1A, compare lanes 2–4 and 7–9). We hypothesized that this was due to the induction of ISG15 at different level in uninfected cells that surround infected cells at low MOIs. To test this hypothesis and to examine the effect of HCMV infection on ISG15 expression at a single cell level, HF cells were infected with HCMV or UV-HCMV at a low MOI (0.5) and stained for ISG15 and viral UL112-113 proteins. We found that ISG15 expression was reduced in HCMV-infected cells, in which UL112-113 viral replication proteins were expressed at high level; however, it was markedly increased in neighboring uninfected cells compared to mock-infected cells. We also found that the levels of ISG15 in uninfected neighboring cells were higher in HCMV infection than in UV-HCMV infection (Fig 1B). This result suggests that an indirect effect of virus infection on neighboring uninfected cells is responsible for the greater induction of ISG15 and protein ISGylation by HCMV than by UV-HCMV at low MOIs.

### HCMV IE1 suppresses ISG15 transcription

**Fig 2:**
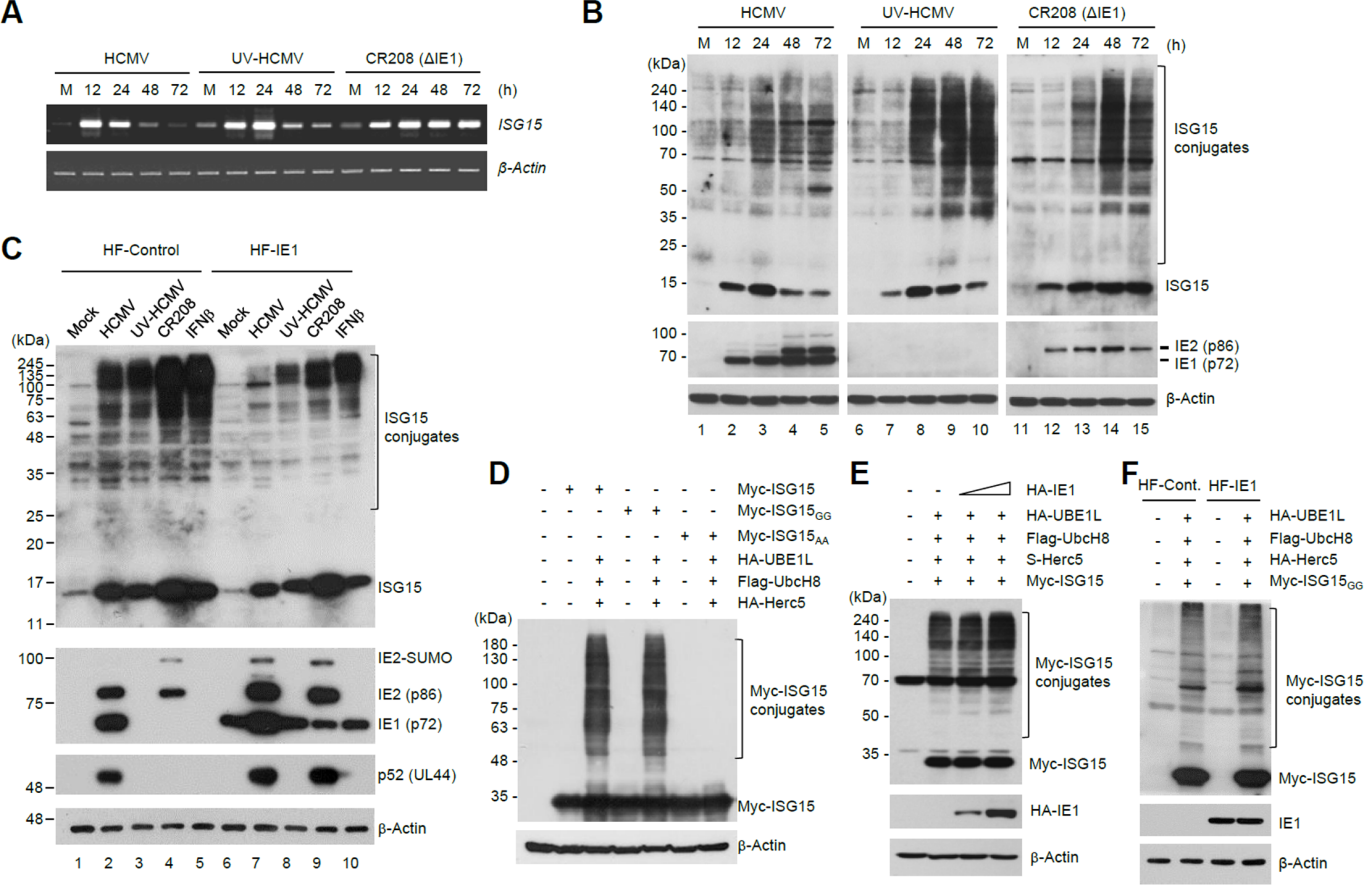
Effects of IE1 on ISG15 transcription and protein ISGylation. (A-B) HF cells were mock-infected (M) or infected with wild-type HCMV, UV-HCMV, or CR208 virus at an MOI of 3. Total RNAs were prepared at the indicated time points and the levels of ISG15 and β-actin transcripts were determined by RT-PCR (A). Cell lysates were also prepared and analyzed by immunoblotting as in Fig 1A (B). (C) Control and IE1-expressing HF cells produced by retroviral vectors were mock-infected or infected with wild-type HCMV, UV-HCMV, or CR208 virus at an MOI of 3, or treated with IFNβ (1,000 U/ml) for 48 h. Immunoblotting was performed with antibodies for ISG15, IE1/IE2, p52 (encoded by UL44) and β-actin. (D) 293T cells were co-transfected with plasmids expressing myc-ISG15 (wild-type, ISG15_GG_, or ISG15_AA_), HA-UBE1L (E1), Flag-UbcH8 (E2), or HA-Herc5 (E3) as indicated. At 48 h after transfection, cell lysates were prepared by boiling the cell pellets in sodium dodecyl sulfate (SDS) loading buffer and immunoblotted with anti-myc and anti-β-actin (a loading control) antibodies. (E and F) Co-transfection/ISGylation assays were performed in 293T cells with or without increasing amounts of plasmid expressing IE1 (E) or in control and IE1-expressing HF cells (F). Immunoblotting was performed with anti-myc, anti-IE1, and anti-β-actin antibodies.

Since HCMV IE1 inhibits the activation of ISRE-containing promoters by sequestering STAT2 [58–60] and PML [61], IE1 expression may be responsible for the suppression of free ISG15 and ISG15 conjugate levels during HCMV infection. To test this hypothesis, we first compared the effects of wild-type HCMV, UV-HCMV, and IE1-deleted mutant virus (CR208) infection (MOI of 3) on ISG15 transcription by RT-PCR. The CR208 virus exhibited an MOI-dependent growth pattern with showing a severe growth defect in HF cells at low MOIs but normal growth at high MOIs [67]. All viruses increased ISG15 mRNA levels 12 h after infection; however, ISG15 induction was terminated earlier for HCMV than UV-HCMV, and not appreciably terminated for CR208, which continued to produce high levels of ISG15 transcripts even at a late stage of infection (72 h) (Fig 2A). ISG15 transcription induced by UV-HCMV infection might be gradually decreased due to the termination of the IFN signaling through several negative regulatory mechanisms. However, when cells were infected with CR208 at this high MOI, the replication of virus without IE1 appeared to lead to a robust activation of IFN signaling. This result indicates that IE1 indeed plays an important role in reducing ISG15 transcription during HCMV infection.

We also compared the levels of ISG15 and ISG15 conjugates in HCMV, UV-HCMV, and CR208 infection. When cells were infected with viruses at an MOI of 3, free ISG15 levels were elevated at 12 h by all viruses, whereas the level of ISG15 conjugates markedly increased after 24 h (Fig 2B). The delayed induction of ISGylation is similar to what was observed in IFNβ-treated cells [68] and may result from the delayed induction of the ISGylation machinery. Consistent with the results shown in Fig 1A, UV-HCMV induced more ISG15 conjugates than wild-type virus at this MOI (Fig 2B, compare lanes 3–5 and 8–10). Importantly, CR208 also resulted in greater ISGylation than wild-type virus (Fig 2C. compare lanes 3–5 and 13–15), indicating that IE1 is required for the suppression of ISG15 expression and ISGylation during HCMV infection. Immunoblot analysis demonstrated that IE1 and IE2 failed to be expressed in UV-HCMV infection and that IE1 failed to be expressed and IE2 levels were reduced in CR208 infection under these experimental conditions (Fig 2B and 2C). Although our results show that IE1 is largely responsible for the suppression of ISG15 transcription, it is notable that CR208 infection resulted in slightly lower levels of ISGylation compared to UV-HCMV infection (Fig 2B, compare lanes 10 and 15). Considering that ISG15 transcript levels remained high up to 72 h in CR208-infected cells (Fig 2A), this finding suggests that other viral processes, besides those mediated by IE1, may also be implicated in the downregulation of protein ISGylation.

The inhibition of ISG15 expression by IE1 was further investigated using IE1-overexpressing HF cells generated using retroviral vectors. Control and IE1-overexpressing HF cells were infected with HCMV, UV-HCMV, or CR208. The results of immunoblot analysis showed that IE1 overexpression suppressed the induction of ISG15 and ISG15 conjugates by both virus infection and IFNβ treatment (as a control) (Fig 2C, compare lanes 2–5 and 7–10), further supporting the critical role of IE1 in reducing ISG15 expression.

Although reduction of protein ISGylation during HCMV infection may be largely attributed to suppression of ISG15 transcription by IE1, it cannot be ruled out that IE1 also affects the ISGylation reaction. Therefore, we studied whether IE1 directly affects ISG15 conjugation reactions using co-transfection/ISGylation assays. To set up co-transfection/ISGylation assays, two different ISG15 forms, an active form with a stop codon immediately after the C-terminal double glycine residues (ISG15_GG_) and an inactive form with the double glycine residues substituted with alanine residues (ISG15_AA_), were employed. In cells transiently co-transfected with ISG15, UBE1L (E1), UbcH8 (E2), and Herc5 (E3), intact ISG15 and ISG15_GG_ (an active form), but not ISG15_AA_ (an inactive form), were conjugated to proteins (Fig 2D). When co-transfection/ISGylation assays were performed with or without IE1 overexpression, IE1 expression by co-transfection or retroviral transduction did not inhibit levels of ISG15 conjugates, indicating that IE1 does not inhibit the enzymatic cascade of reactions required for protein ISGylation (Fig 2E and 2F).

### ISGylation inhibits HCMV growth

**Fig 3:**
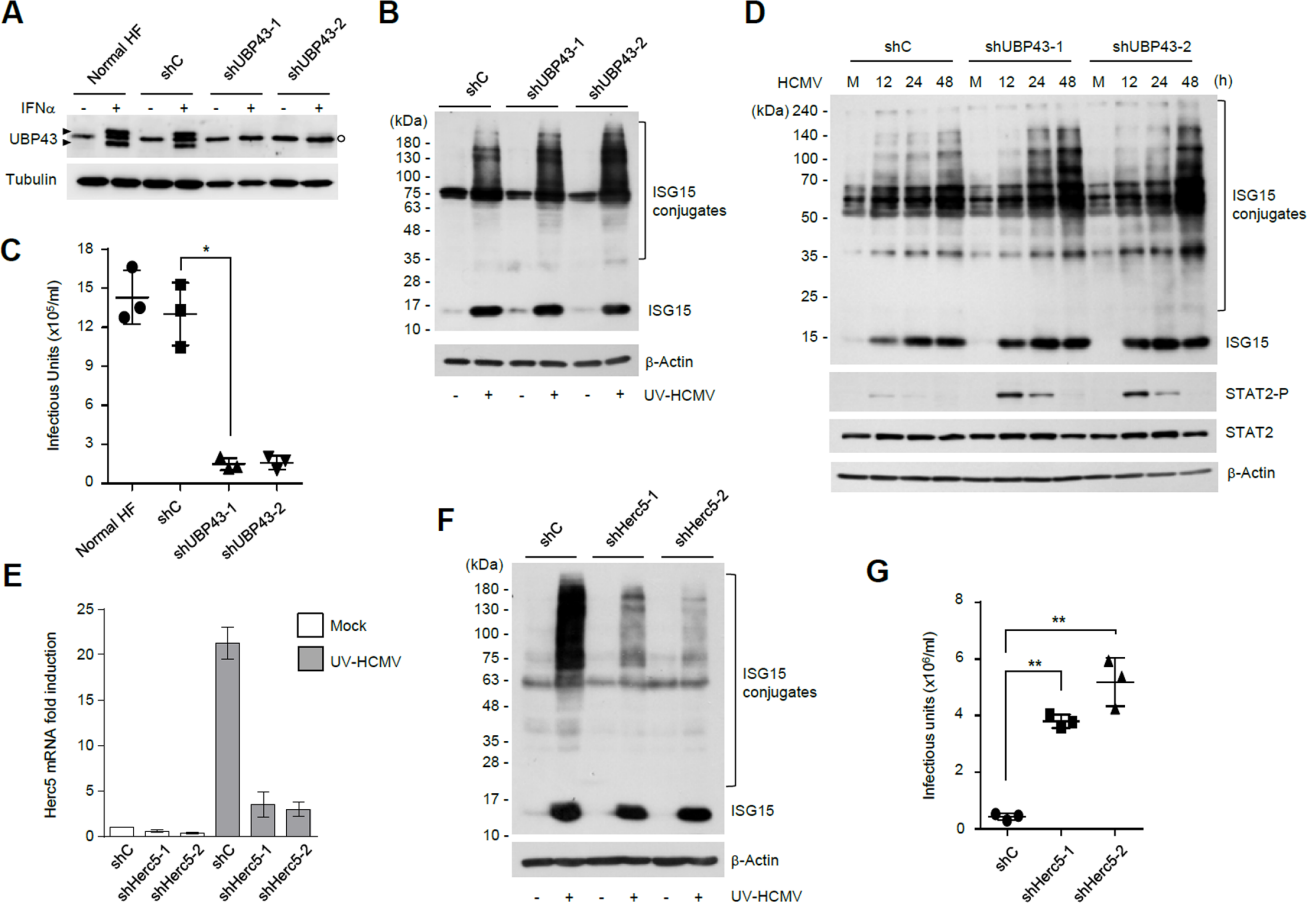
Effects of UBP43 or Herc5 knockdown on HCMV growth. (A) Normal HF cells or cells expressing control (shC) or UBP43-specific shRNAs (shUBP43-1 and -2) were treated with IFNα (2,000 U/ml) for 24 h and the UBP43 levels were analyzed by immunoblotting using anti-UBP43 antibody. The tubulin levels were shown as a loading control. Arrowheads indicate two UBP43 bands with different molecular weights and an open circle indicates non-specific bands. (B) Control and UBP43 knockdown HF cells were infected or not with UV-HCMV at an MOI of 3. At 24 h after infection, cell lysates were immunoblotted with antibodies for ISG15 and β-actin. (C) Control and UBP43-knockdown HF cells were infected with HCMV at an MOI of 3. At 5 days after infection, viral supernatants were collected and the levels of progeny virions were measured by infectious center assays. Scatter plots are shown. (D) Control and UBP43-knockdown cells were infected as in (C). Cell lysates were prepared at indicated time points and immunoblotted with antibodies for ISG15, STAT2, phosphorylated STAT2 (on Tyr 689), and β-actin. (E) Control HF cells or cells expressing Herc5-specific shRNA were mock-infected or infected with UV-HCMV at an MOI of 3. The Herc5 transcript levels were determined at 24 h after infection by qRT-PCR. The β-actin mRNA levels were used for normalization. Values are an average of duplicated assays; error ranges are indicated. (F) Control and Herc5-knockdown HF cells were infected or not with UV-HCMV at an MOI of 3. At 24 h after infection, cell lysates were immunoblotted with antibodies for ISG15 and β-actin. (G) Control and Herc5-knockdown HF cells were infected with HCMV at an MOI of 0.1. At 9 days after infection, virus titers in the culture supernatants were measured by infectious center assays.

The role of protein ISGylation in HCMV infection was investigated by silencing expression of ISGylation enzymes using RNA interference. HF cells expressing control shRNA or shRNA for UBP43, an ISG15-specific protease, were generated using lentiviral vectors. IFNα treatment of normal and control shRNA (shC)-expressing HF cells induced the expression of UBP43 proteins; however, UBP43-specific shRNAs (shUBP43-1 and shUBP43-2) efficiently suppressed this induction (Fig 3A). UBP43 knockdown enhanced protein ISGylation in cells infected with UV-HCMV that stimulates interferon signaling (Fig 3B), but reduced the production of progeny virions in HCMV-infected cells to nearly 10% of that in control cells (Fig 3C), suggesting that enhanced ISGylation by UBP43 knockdown attenuates HCMV growth. However, UBP43 is also known to negatively regulate IFN signaling by downregulating JAK/STAT signaling [15, 69]. Consistently, we found that UBP43 knockdown enhanced STAT2 phosphorylation in HCMV-infected cells (Fig 3D). Therefore, it is possible that the reduction of HCMV growth in UBP43 knockdown cells is also a result of other aspects of the IFN response.

The effect of ISGylation on HCMV growth was further assessed by depleting Herc5, a major ISG15 E3 ligase in human [6, 7]. Herc5 knockdown HF cells were generated using lentiviral vectors expressing shRNAs. qRT-PCR assays confirmed the efficient reduction of Herc5 transcript levels in cells expressing Herc5-specific shRNAs (shHerc5-1 and shHerc5-2) compared to in cells expressing control shRNA (shC) after UV-HCMV infection (Fig 3E). We found that Herc5 knockdown markedly reduced protein ISGylation as expected (Fig 3F), but increased virus titers by 8- to 11-fold compared to in control cells (Fig 3G), demonstrating that the reduction of ISGylation by Herc5 knockdown facilitates HCMV growth. Similar enhancement of HCMV growth was observed in HF cells depleted of UBE1L (E1) or UbcH8 (E2) (S1 Fig). Collectively, our results with UBP43, Herc5, UBE1L, and UbcH8 knockdown cells demonstrate an inverse relationship between ISGylation and HCMV growth, indicating a general inhibitory role of ISGylation in HCMV infection.

We also investigated whether enhanced ISGylation affects HCMV growth by ectopically expressing ISG15 together with ISGylation enzymes. HF cells co-transfected with plasmids expressing myc-ISG15_GG_ or myc-ISG15_AA_ and ISGylation enzymes were infected with HCMV and the production of progeny virions was compared. The results showed that the expression of myc-ISG15_GG_ and ISGylation enzymes induced higher levels of ISG15 conjugates and reduced progeny virus titers to 50% of control, whereas the expression of ISG15_AA_ did not significantly affect the levels of ISG15 conjugates or progeny virions (S2 Fig). These results are also consistent with our results using shRNA-expressing cells in which we showed an inverse relationship between levels of ISG15 conjugates and HCMV growth.

### ISGylation reduces HCMV gene expression and virion release

**Fig 4:**
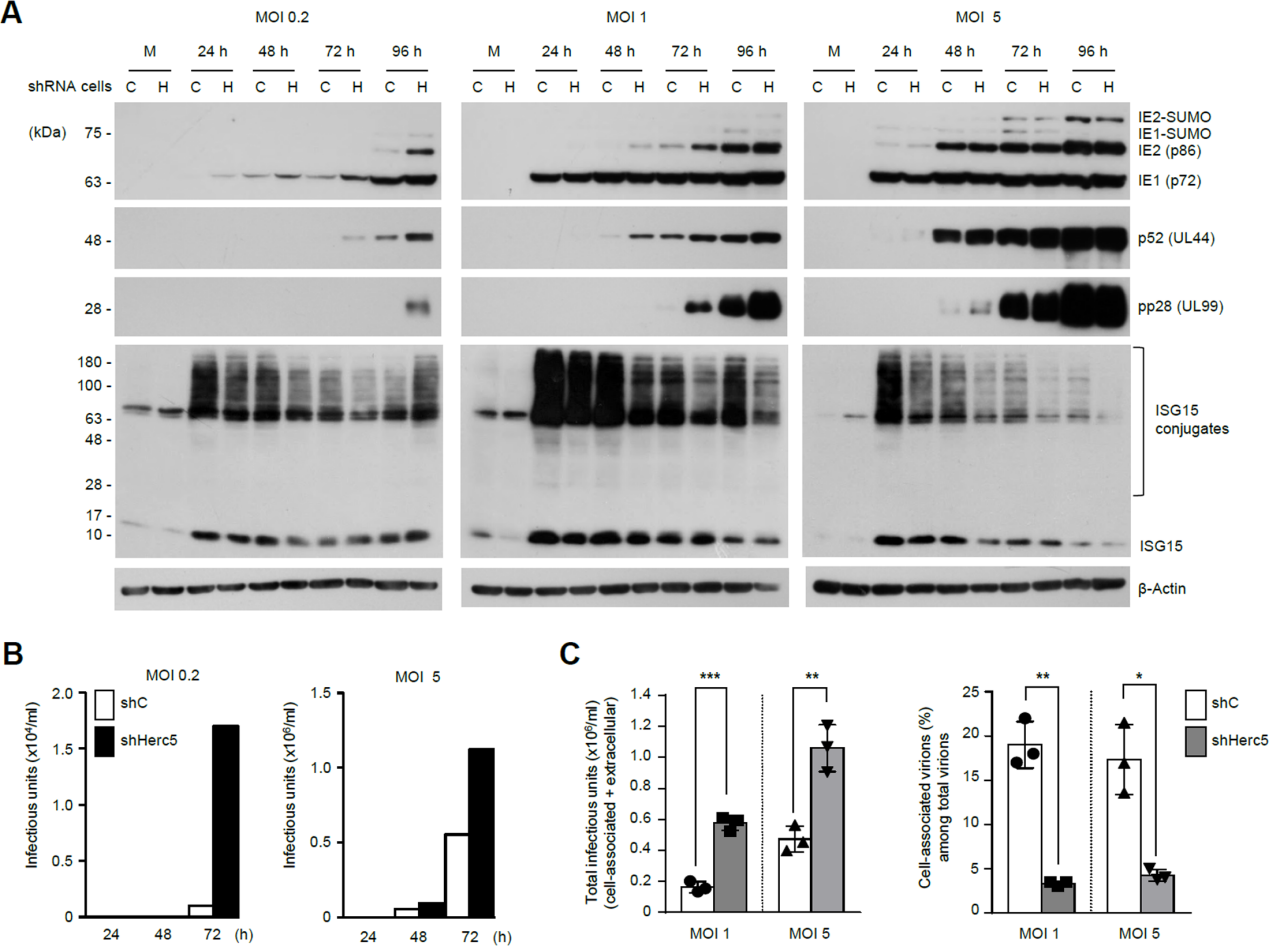
Effect of Herc5-knockdown on HCMV gene expression and virion release. (A-B) Control HF cells expressing shC (C) and Herc5-knockdown cells expressing shHerc5-2 (H) were mock-infected or infected with HCMV at an MOI of 0.2, 1, or 5. Cell lysates were immunoblotted at the indicated time points with antibodies for IE1/IE2, p52, pp28 (encoded by UL99), ISG15, and β-actin (A). Virus titers produced in the culture supernatants for 24, 48, and 72 h were determined by infectious center assays (B). (C) Control (shC) and Herc5-knockdown HF cells were infected with HCMV at an MOI of 1 or 5. At 4 days after infection, virus titers produced within the cells (cell-associated) and in the culture supernatants (extracellular) were determined by infectious center assays. The total amounts of infectious units (cell-associated plus extracellular) produced in control and Herc5-knockdown cells were shown at the top. The percentages of cell-associated virions among total virions in control or Herc5-knockdown cells were shown at the bottom. The results are the mean values of three independent experiments with standard errors.

To investigate the mechanisms by which ISGylation inhibits HCMV growth, we first compared the expression profile of viral proteins in control and Herc5 knockdown cells. When cells were infected with an MOI of 0.2, levels of major IE (IE1 and IE2), early (p52), and late (pp28) viral proteins produced at 24, 48, 72, and 96 h were higher in Herc5 knockdown cells than in control cells (Fig 4A, left panels). This effect of knocking down Herc5 diminished when cells were infected at higher MOIs. At an MOI of 1, IE1 expression was similar between control and Herc5 knockdown cells, although the levels of IE2, p52, and pp28 were slightly increased in Herc5 knockdown cells (Fig 4A, center panels). At an MOI of 5, levels of viral proteins were comparable between control and Herc5 knockdown cells (Fig 4A, right panels). Progeny virion titers measured in the culture supernatant correlated with the levels of expressed viral proteins (Fig 4B). These results demonstrate that reduced ISGylation by silencing Herc5 promotes viral gene expression, an effect that is more evident at lower MOIs.

We further investigated whether ISGylation affects the activation of viral promoters using reporter assays (S3 Fig). HF cells co-transfected with ISG15, UBE1L, UbcH8, and Herc5 exhibited substantially increased protein ISGylation, whereas cells co-transfected without Herc5 did not. In HF cells exhibiting enhanced ISGylation by co-transfection, the activity of viral MIE promoter was repressed to 50% of that observed in control cells. Similarly, IE2-mediated activation of viral early [UL112-113 and UL54 (POL)] and late [UL99 (pp28)] promoters was also suppressed in cells showing enhanced ISGylation. In control experiments, ectopic expression of Herc5 alone was not sufficient to increase ISGylation and therefore did not affect viral promoter activation. These results demonstrate that enhanced ISGylation inhibits the activation of viral promoters.

We also assessed whether ISGylation inhibits HCMV virion release as is the case in HIV infection [43]. Control and Herc5 knockdown cells were infected with HCMV at an MOI of 1 or 5 and the levels of cell-associated and extracellular progeny virions produced were compared. The results showed that although more progeny virions were produced in Herc5 knockdown cells than in control cells (Fig 4B), the percentages of cell-associated virions to total virions were lower in Herc5-knockdown cells, 3.5% (MOI 1) and 3.8% (MOI 5), than in control cells, 19.7% (MOI 1) and 17% (MOI 5) (Fig 4C). These results indicate that, as observed in HIV infection, ISGylation inhibits HCMV virion release.

### Covalent and non-covalent interaction of pUL26 with ISG15

**Fig 5:**
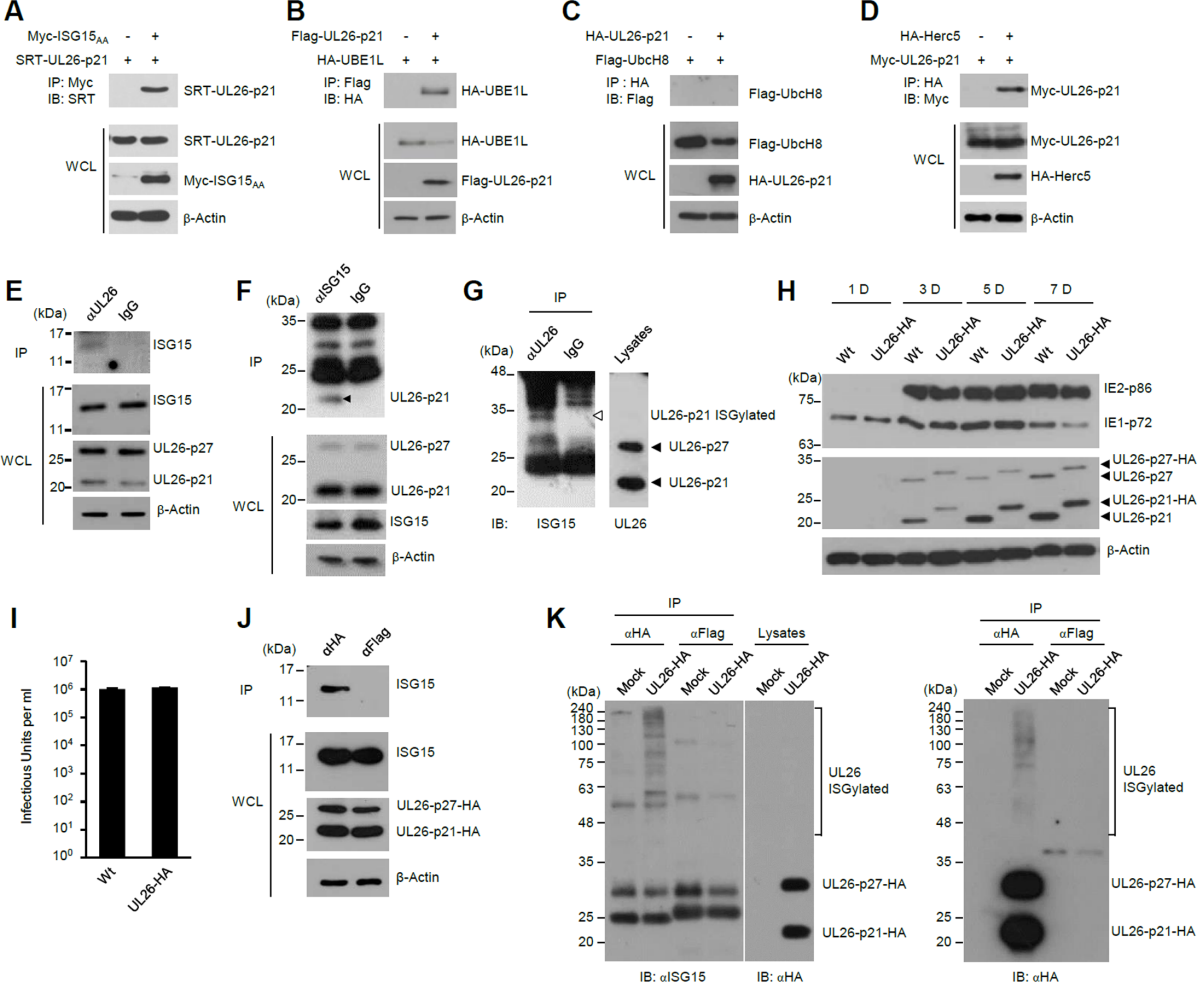
Interactions of pUL26 with ISG15, UBE1L, and Herc5, and ISGylation of pUL26. (A-D) 293T cells were co-transfected with plasmids encoding myc-ISG15_AA_, HA-UBE1L, Flag-UbcH8, HA-Herc5, SRT-UL26-p21, Flag-UL26-p21, or HA-UL26-p21, as indicated. At 48 h after transfection, cell lysates were prepared and immunoprecipitated with anti-myc (A), anti-flag (B), or anti-HA (C and D) antibodies, followed by immunoblotting with anti-SRT (A), anti-HA (B), anti-flag (C), or anti-myc antibodies. To determine the expression levels of each protein, whole cell lysate were also immunoblotted. (E and F) HF cells were infected with HCMV(Towne) at an MOI of 3. At 48 h after infection, cell lysates were subjected to co-IP assays using anti-UL26 antibody and control IgG (E) or using anti-ISG15 antibody and control IgG (F), followed by immunoblotting with anti-ISG15 (E) or anti-UL26 (F) antibodies. Immunoblotting of whole cell lysates were performed to determine the expression levels of each protein. (G) HF cells were infected as in (E) for 48 h. Cell lysates were prepared and immunoprecipitated with anti-UL26 antibody and control IgG as described for co-IP assays to detect ISGylated protein (see Materials and Methods). Immunoprecipitated samples and total cell lysates were immunoblotted with anti-ISG15 and anti-UL26 antibodies. (H and I) HF cells were infected with wild-type HCMV (Toledo) or its recombinant virus expressing UL26-HA proteins at an MOI of 3. Cell lysates were prepared at the indicated time points and immunoblotted with anti-IE1/IE2, anti-HA, and anti-β-actin antibodies (H). The culture supernatants were collected at 3 days after infection and virus titers were determined by infectious center assays (I). (J) HF cells were mock-infected or infected with recombinant virus expressing UL26-HA at an MOI of 3 for 48 h. Cell lysates were subjected to co-IP assays using anti-HA antibody and anti-Flag antibody, followed by immunoblotting with anti-ISG15 antibody as in (G). Immunoblotting of whole cell lysates were performed to determine the expression levels of each protein. (K) HF cells were mock-infected or infected as in (J). Cell lysates were prepared and immunoprecipitated with anti-HA antibody as in (G). Immunoprecipitated samples and total cell lysates were immunoblotted with anti-ISG15 and anti-HA antibodies as indicated.

To identify potential HCMV proteins that interact with the ISG15 pathway, we screened the HCMV ORF library [70] for ISG15- or UBE1L-interacting proteins using yeast two-hybrid assays. Twenty HCMV proteins were identified as potential ISG15-interacting proteins, and five viral proteins (encoded from RL1, UL19, UL21A, UL26, and UL30) were found to interact with both ISG15 and UBE1L. Most of these ISG15 binding and all of UBE1L binding in yeast assays were also detected by co-immunoprecipitation (co-IP) assays (S4 Fig and S2 Table). Among them, the UL26 gene encodes the tegument proteins, p27 and p21, which are produced using two in-frame start codons and are shown to regulate viral gene expression, NF-κB signaling, and virion stability [71–74]. Since UL26 interacted with both ISG15 and UBE1L and its role in viral growth was relatively well reported compared to others, we further investigated the interaction of pUL26 with the ISG15 pathway. In co-IP assays, UL26-p21 interacted with ISG15_AA_, demonstrating that ISG15 can non-covalently interact with UL26 (Fig 5A). In similar co-IP assays, UL26-p21 also interacted with UBE1L and Herc5, but not UbcH8 (Fig 5B–5D). When co-IP assays were performed using HCMV (Towne)-infected cell lysates, immunoprecipitation with an anti-UL26 antibody co-precipitated unconjugated free ISG15 (Fig 5E), and in a reciprocal experiment, immunoprecipitation with an anti-ISG15 antibody co-precipitated UL26-p21 (Fig 5F), indicating that UL26-p21 interacts with ISG15 during virus infection.

It has been suggested that newly synthesized viral proteins may be broadly modified by ISG15 during virus infection [41]. Therefore, we also investigated whether UL26 is covalently conjugated by ISG15 during HCMV infection. Cell lysates prepared from HCMV (Towne)-infected cells were boiled in SDS-containing buffer and then immunoprecipitated with an anti-UL26 antibody. Immunoblotting of the sample with anti-ISG15 antibody revealed a band that is consistent with an ISG15-modified form of UL26-p21 (Fig 5G). To further investigate these non-covalent and covalent interaction of UL26 proteins with ISG15, we generated a recombinant HCMV (Toledo) expressing UL26 proteins tagged at their carboxyl termini with an HA tag (S5 Fig). Compared to the wild-type virus, the recombinant virus produced equivalent amounts of viral major IE (IE1 and IE2) and UL26 proteins, and progeny virions (Fig 5H and 5I). When co-IP assays were performed with lysates from UL26-HA virus infected cells, immunoprecipitation of UL26 proteins with an anti-HA antibody co-precipitated free ISG15 (Fig 5J). When HA-UL26 virus-infected cells were subjected to co-IP assays to detect ISGylated proteins, bands that are consistent with ISG15-modified forms of UL26 proteins were detected (Fig 5K). Since ISG15 can modify ubiquitin, forming ISG15-ubiquitin mixed chains [75] and a single lysine reside can be poly-ISGylated [20, 76], the smear bands of ISGylated UL26 appear to be UL26 proteins that contain ISG15-ubiquitin mixed chains or poly-ISG15 chains. Taken together, our data suggest that the UL26-encoded proteins non-covalently interact with ISG15 and are also covalently modified by ISG15. In a control experiment, we found that some viral proteins, which did not interact with ISG15, UBE1L, and Herc5 in co-IP assays, could be ISGylated in co-transfection/ISGylation assays (S6 Fig), supporting the concept that viral proteins may be broadly ISGylated during infection due to IFN-upregulated expression of Herc5 (E3) in polyribosomes [41].

### ISGylation of UL26 regulates protein stability and inhibits UL26 activities to suppress NF-κB signaling and promote viral growth

**Fig 6:**
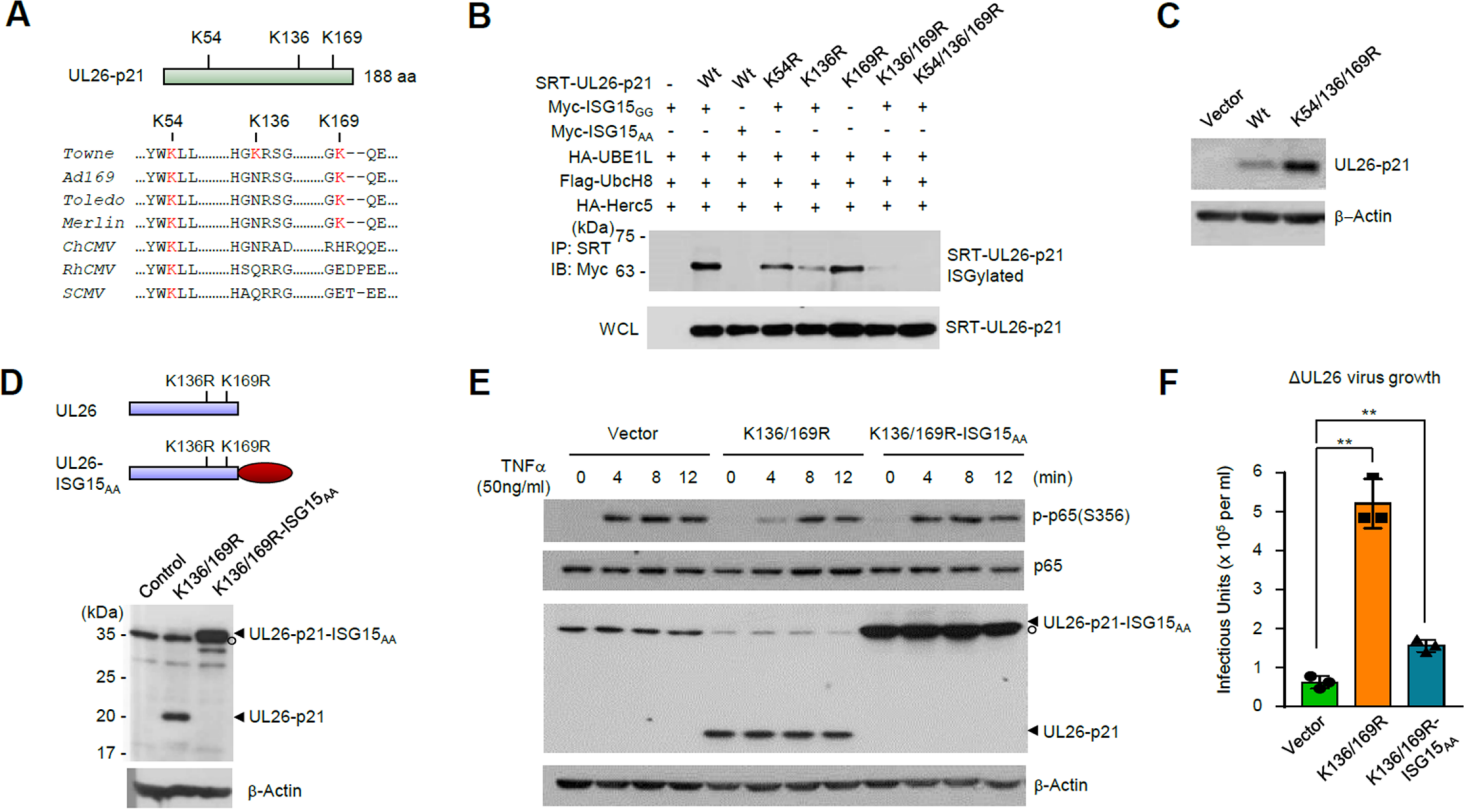
Roles of UL26 ISGylation in viral growth. (A) Three lysine residues of UL26-p21 from Towne strain and their conservation in other human (Toledo, Ad169, and Merlin) and primate CMVs (chimpanzee CMV, ChCMV; rhesus CMV, RhCMV; simian CMV, SCMV) are shown. (B) 293T cells were co-transfected with plasmid expressing SRT-UL26-p21 (wild-type or lysine to arginine mutants), myc-ISG15 (with GG or AA terminus), HA-UBE1L, Flag-UbcH8, or HA-Herc5 as indicated. At 48 h after transfection, cell lysates were immunoprecipitated with anti-SRT antibody. Immunoprecipitated samples and whole cell lysates were detected by immunoblotting with anti-myc and anti-SRT antibodies. (C) Control HF cells or cells expressing UL26-p21 (wild-type or K136/359R mutant) were produced by retroviral vectors. Cell lysates were prepared and immunoblotted with antibodies for UL26 and β-actin (a loading control). (D-F) Control HF cells or cells expressing UL26-p21(K136/169R) or UL26-p21(K136/169R)-ISG15_AA_ were produced by retroviral transduction. To determine the expression levels of UL26 proteins, cell lysates were immunoblotted with antibodies for IE1/IE2, UL26, and β-actin (D). Cells were treated or not with 50 ng/ml of TNFα for the indicated times. Immunoblotting was performed to detect the levels of p65 and its phosphorylated form. The levels of UL26-p21, its ISG15_AA_-fused form, and β-actin were also shown (E). Cells were infected with the UL26-deleted mutant virus (AD169) at an MOI of 0.2. At 72 h after infection, virus titers in the culture supernatants were determined by infectious center assays (F).

The UL26 ORF from the Towne strain contains three lysine residues (K54, K136, and K169) and K54 and K169 are conserved in human CMVs (Fig 6A). To determine the ISGylation sites of UL26 proteins, we performed co-transfection/ISGylation assays using wild-type UL26-p21 or its mutants in which lysine residues were replaced with arginines. The results showed that the K54R, K136R, and K169R mutants were still ISGylated; however, the K136/169R double mutant was not ISGylated, indicating that K136 and K169 are the major ISGylation sites (Fig 6B). When HF cells expressing wild-type UL26-p21 or K136/169R mutant were generated by retroviral vectors, expression level of the K136/169R mutant protein was higher than that of the wild-type protein (Fig 6C), suggesting that ISGylation of UL26 proteins may regulate protein stability.

The SUMO fusion proteins were often used to study the function of SUMO modification of proteins [77–81]. To investigate the effect of UL26 ISGylation on its activity, we used the UL26-p21(K136/169R)-ISG15_AA_ fusion protein as a surrogate for the ISG15-modified form of UL26-p21. We produced the ISG15 fusion to the lysine mutant form of UL26 to compare the activities of ISGylation-defective UL26 and its ISG15 fusion form. HF cells expressing the K136/169R mutant or the UL26-p21(K136/169R)-ISG15_AA_ fusion protein were generated by retroviral vectors (Fig 6D). UL26 proteins have been shown to inhibit TNFα-induced NF-κB activation [74]. UL26-p21(K136/169R) moderately inhibited TNFα-induced NF-κB activation but UL26-p21(K136/169R)-ISG15_AA_ did not, suggesting that ISGylation of UL26 inhibits its activity to downregulate NF-κB signaling (Fig 6E). We further investigated the effect of UL26 ISGylation on its role in viral growth. When control and UL26-expressing cells were infected with the UL26-deleted mutant HCMV (AD169) [72] and the levels of progeny virus titers were compared, pre-expression of the K136/169R mutant significantly increased the growth of mutant virus but its ISG15_AA_ fusion protein did not (Fig 6F). Together, these results indicate that ISGylation of UL26 inhibits its activities to suppress NF-κB signaling and promote the growth of UL26-deleted mutant virus.

### UL26 inhibits protein ISGylation

**Fig 7:**
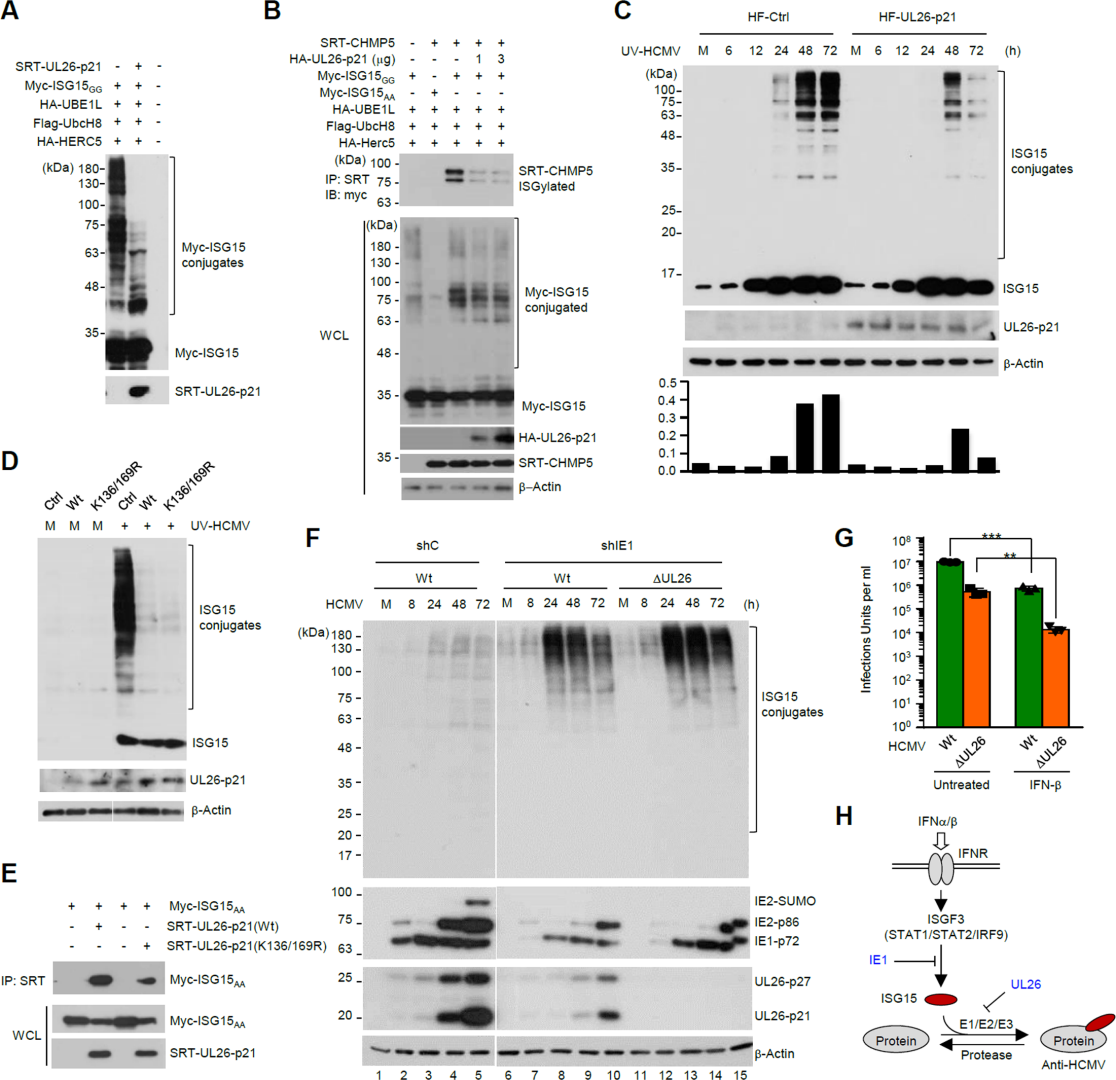
Inhibition of protein ISGylation by UL26. (A) 293T cells were co-transfected with expression plasmids as indicated. At 48 h after transfection, cell lysates were immunoblotted with anti-myc and anti-SRT antibodies. (B) 293T cells were co-transfected with expression plasmids as indicated. At 48 h after transfection, cell lysates were prepared and immunoprecipitated with anti-SRT antibody, followed by immunoblotting with anti-myc antibody. Expression levels of myc-ISG15, SRT-CHMP5, and β-actin in whole cell lysates were also determined by immunoblotting. (C) Control and UL26-p21-expressing HF cells produced by retroviral vectors were infected with UV-HCMV at an MOI of 1. The samples were prepared at the indicated time points and the levels of ISG15, UL26 and β-actin were determined by immunoblotting. The relative levels of ISGylated UL26 protein over unmodified protein (normalized with β-actin) are also shown as graphs. (D) Control or UL26 (wild-type or mutant)-expressing HF cells were infected with UV-HCMV and immunoblotting was performed as described in (C). (E) 293T cells were co-transfected with plasmids expressing SRT-UL26-p21 (wild-type or K136/169R) or myc-ISG15_AA_ as indicated. At 48 h after transfection, cell lysates were immunoprecipitated with anti-SRT antibody. Immunoprecipitated samples and whole cell lysates were detected by immunoblotting. (F) HF cells expressing control shRNA (shC) or shRNA specific for IE1 (shIE1) produced by retroviral transduction were infected with wild-type or ΔUL26 mutant virus at an MOI of 5 for the indicated time points. Cell lysates were immunoblotted with antibodies for ISG15, viral proteins (IE1, IE2, and UL26), and β-actin. (G) HF cells were pre-treated or not with IFNβ (100 U/ml) for 24 h and then infected with wild-type or ΔUL26 mutant virus at an MOI of 1. The production of progeny virions in the culture supernatant at 6 days after infection was measured by infectious center assays. The results shown are the mean values and standard errors of four independent experiments. (H) Summary for the HCMV strategy that consecutively inhibits ISG15 transcription and protein ISGylation. IE1 inhibits ISG transcription, while UL26 inhibits protein ISGylation.

Since influenza virus NS1B antagonizes ISGylation in human cells via direct binding to ISG15 [3, 82], we tested whether UL26 can regulate protein ISGylation. We found that expression of UL26-p21 reduced the levels of ISG15 conjugates in cells co-transfected with UBE1L (E1), UbcH8 (E2), Herc5 (E3), and ISG15_GG_, an active form of ISG15 (Fig 7A). In a similar assay, ISGylation of charged multivesicular body protein (CHMP) 5, a component of the endosomal sorting complex required for transport (ESCRT) machinery, was inhibited by UL26-p21 expression (Fig 7B). When control and UL26-p21-expressing HF cells were infected with UV-HCMV, the levels of ISG15 conjugates were significantly reduced in UL26-p21-expressing cells compared to control cells (Fig 7C). This effect of UL26 was also found with the K136/169R mutant protein (Fig 7D), which still interacted with ISG15_AA_ in co-IP assays (Fig 7E), suggesting that the inhibitory effect of UL26 on ISGylation is not dependent on its own ISGylation. In control experiments, viral proteins such as pUL85, pUL71, and IE2 did not inhibit ISGylation of cellular proteins in co-transfection/ISGylation assays (S6 and S7 Figs).

To further investigate the inhibitory effect of UL26 on ISGylation during HCMV infection, we compared ISGylation levels between cells infected with wild-type and UL26-deleted mutant viruses. With an MOI of 0.2, we found that the ISGylation levels were initially increased until 48 h but gradually decreased at 72 h and 96 h, and that the UL26-deleted virus infection showed only minimally increased levels of ISGylation at late times of infection compared to wild-type virus infection (S8 Fig, compare lanes 5–7 and 11–13). We reasoned this minimal effect of UL26 to the suppression of ISG15 transcription by IE1. Therefore, to minimize the effect of IE1 expression we performed a similar experiment in cells expressing shRNA for IE1 (shIE1). Control shRNA (shC) and shIE1-expressing HF cells, which were produced by retroviral vectors, were infected with wild-type and UL26-deleted mutant viruses at an MOI of 5. The results of immunoblotting showed that the expression of shIE1 substantially reduced the IE1 protein accumulation compared to control cells (Fig 7F, compare lanes 2–5 and 7–10), and that in shIE1-expressing cells the ISGylation levels were substantially increased at 24 h after infection and gradually decreased at 48 h and 72 h under these experimental conditions (Fig 7F, lanes 8–10). Notably, we found that the UL26-deleted virus less effectively reduced the ISGylation levels than wild-type virus at the late phase of infection (Fig 7F, compare lanes 8–10 and 13–15). These results demonstrate an inhibitory effect of UL26 on ISGylation during virus infection.

Since HCMV growth was suppressed by IFN-induced ISGylation and UL26 inhibited ISGylation, we tested whether UL26-deleted mutant virus is more susceptible to type I IFN treatment than wild-type virus. We found that while the titers of wild-type virus were reduced by 12-fold by IFNβ pre-treatment, those of UL26-deleted virus were reduced by 40-fold, indicating that UL26-deleted virus less effectively overcomes the IFNβ-mediated anti-viral responses than wild-type virus (Fig 7G). Overall, our results demonstrate that, in addition to IE1 that suppresses ISG15 transcription, UL26 plays an important role in evading the ISG15-associated antiviral responses by inhibiting protein ISGylation (Fig 7H).

## Discussion

Our analysis with UV-HCMV and IE1-deleted mutant virus demonstrates that ISGylation is induced by HCMV infection and that IE1 plays a central role in downregulating ISGylation by reducing ISG15 transcription. The latter is consistent with previous findings that IE1 represses transcription of ISGs by sequestrating STAT2 [58–60] and PML [61, 62]. Notably, although IE1 effectively reduced ISG expression, the level of ISGylation during HCMV infection largely depended on the MOI.

Our data provide evidence for the antiviral roles of ISGylation during HCMV infection. To discern the effects of free ISG15 expression and protein ISGylation on virus replication, we used HF cells in which a specific ISGylation enzyme was depleted by shRNA. The data consistently showed an inverse relationship between the level of ISG15 conjugates and HCMV growth; i.e., enhanced ISGylation by UBP43 knockdown decreased viral growth, while reduced ISGylation by depletion of UBE1L, UbcH8, or Herc5 increased viral growth. Therefore, we conclude that protein ISGylation in general inhibits HCMV growth. Free ISG15 has also been shown to inhibit the replication of certain viruses [34, 35, 44]. In our analysis, however, overexpression of ISG15_GG_ with UBE1L, UbcH8, and Herc5 led to a mild reduction of HCMV growth, whereas ISG15_AA_, an inactive form, did not significantly affect viral growth, suggesting that expression of free ISG15 prior to HCMV infection may minimally affect viral growth in cultured cells. It should be noted that given the role of free ISG15 in stabilizing UBP43 (or USP18), a negative regulator of IFN signaling [53], overexpression or depletion of ISG15 might affect both positive and negative activities of ISG15 to viral growth.

HCMV replication was inhibited by ISGylation at multiple steps. First, the expression of viral genes was inhibited under conditions where the level of ISG15 conjugates was increased. Viral regulators that promote viral gene expression may be a direct target of ISGylation. However, we could not observe ISGylation of IE1, which is responsible for the activation of MIE promoters, or IE2, a strong transactivator of viral early and late genes. ISGylation of cellular proteins that in particular play a role in innate immune responses may affect viral gene expression. Notably, ISGylation of IRF3 increases its stability and enhances IRF3-mediated transcriptional activation during Sendai virus infection [16, 21] and ISGylation of 4EHP, an mRNA 5' cap structure-binding translation suppressor, plays a role in IFN-induced innate immune response [9]. Second, HCMV virion release was inhibited by ISG15 conjugation. The inhibitory role of ISG15 expression in the budding process of enveloped viruses has been demonstrated in retrovirus infection. ISG15 expression inhibits ubiquitination of HIV Gag and tumor susceptibility gene-101 (Tsg101) proteins, leading to disruption of their interaction [26]. ISG15 conjugation of CHMP5, 2A, and 6 in the ESCRT machinery causes release of vacuolar protein-sorting 4 (VPS4) from the membrane, leading to inhibition of virion release [43]. The ESCRT machinery seems to be involved in the process of HSV-1 and HCMV maturation [83–86]. Therefore, ISGylation may affect the HCMV maturation process by targeting components involved in the ESCRT machinery.

Although the antiviral role of ISG15 expression and ISGylation has been demonstrated in several viruses, studies on viral targets for ISGylation and viral strategies that interfere with the ISG15-mediated antiviral functions are limited to a few examples. ISGylation of NS1A of influenza A virus inhibits virus replication by interfering with NS1A nuclear import [42]. KSHV vIRF1 is ISGylated but it role on vIRF1 function is not known [39]. In the present study, we demonstrated that HCMV pUL26 is a target for ISGylation. UL26 ISGylation appears to regulate protein stability by competing with ubiquitination. Our analysis using the UL26-p21-ISG15 fusion protein demonstrated that ISGylation inhibits the activities of UL26-p21 to downregulate the TNFα-mediated NF-κB activation and to complement the growth defect of UL26-deleted virus. Therefore, ISGylation of pUL26 is thought to inactivate its function, suppressing HCMV growth. Given the notion that newly synthesized proteins are targeted extremely broadly, perhaps stochastically, by Herc5 in polyribosomes [41], several other HCMV proteins may be ISGylated during infection. We indeed observed that some viral proteins, which did not interact with ISG15, UBE1L, and Herc5 in co-IP assays, were ISGylated in our co-transfection/ISGylation assays. However, we think that ISGylation occurs in a manner dependent on the context of each protein. Furthermore, since several HCMV proteins were bound to ISG15 and/or UBE1L in yeast and co-IP assays, it is also likely that other viral proteins besides pUL26 may affect ISGylation. Identification of more HCMV proteins that interact with the ISGylation system and studies on their functional relevance are warranted.

A few examples for viral regulation of ISGylation have been described. The NS1 protein of influenza B virus non-covalently binds to ISG15 and inhibits ISGylation [3]. Similarly, the vaccinia virus E3 protein interacts with ISG15 and disrupts its antiviral activity [36]. Nairoviruses and arteriviruses have shown to encode ovarian tumor domain (OTU)-containing proteases that hydrolyze ISG15 from target proteins [87] and severe acute respiratory syndrome (SARS) coronavirus encoded the papain-like protease that cleaves both ubiquitin and ISG15 [88]. Recently, KSHV-encoded vIRF1 was shown to interact with Herc5 [39]. In this study, we demonstrated that HCMV UL26-p21 is able to non-covalently bind to ISG15, UBE1L, and Herc5, and inhibit ISGylation. Whether binding all of ISG15, UBE1L, and Herc5 is critical for pUL26 to inhibit ISGylation is not clear and needs to be further investigated. Comparative analysis of wild-type and UL26-deleted mutant viruses demonstrate that *de novo* expression of UL26 in virus-infected cells correlated with reduced accumulation of ISG15 conjugates. In addition to IE1, the presence of additional viral functions that downregulate protein ISGylation was prompted by our observation that UV-HCMV infection resulted in a higher level of ISG15 conjugates than IE1-deleted mutant virus at late times (Fig 2B). UL26 deletion mutant virus shows a moderate growth defect at low MOIs [72, 89]. Notably, the UL26 virus (in AD169 strain) showed a reduced plaque formation at low MOIs and this defect could be rescued by IE1 overexpression [72]. More importantly, like IE1-deleted virus [58–60], the growth of UL26-deleted virus was more sensitive to pre-treatment of type I IFNs [74] (and this study), suggesting a role of UL26 in antagonizing type I IFN response. It is likely that a common ISG15-targeting mechanism is shared by influenza B virus, vaccinia virus, and HCMV. HCMV also encodes a tegument protein pUL48 that contains a deubiquitinating protease domain; however, its activity was specific for ubiquitin and did not cleave ISG15 [90].

In this study, we demonstrate that the cellular ISGylation system is a critical part of cellular innate immune response against HCMV infection. We also provide evidence that HCMV has developed several strategies to disarm the ISGylation-mediated antiviral activity; IE1 reduces ISG15 transcription and UL26 inhibits protein ISGylation. This interplay of HCMV with the cellular ISGylation system may be critical for the virus to successfully establish a persistent infection.

## Materials and Methods

### Cell culture, transfection, and virus

Human foreskin fibroblast (HF) (ATCC) and human embryonic kidney (HEK) 293T cells (ATCC) were grown in Dulbecco's modified Eagle's medium supplemented with 10% fetal bovine serum, penicillin (100 U/ml), and streptomycin (100 μg/ml). DNA transfection of 293T cells was performed using the *N*,*N*-bis-(2-hydroxyethyl)-2-aminoethanesulfonic acid-buffered saline (BBS) version of the calcium phosphate procedure. Electroporation of HF cells was conducted using a Microporator MP-100 (Digital Bio), as described previously [59]. Stocks for the parent Towne virus and the CR208 mutant virus with IE1-deleted were prepared in ihfie1.3 cells as described previously [59]. The HCMV (Towne strain)-GFP virus was grown in HF cells after electroporation with the bacmid DNAs. Wild-type and UL26-deleted HCMVs (AD169 strain) were previously described [72] and grown in UL26-expressing HF cells. Wild-type and UL26-HA-expressing HCMVs (Toledo strain) generated in this study were grown in HF cells. To produce UV-inactivated HCMV (UV-HCMV), the virus stock was irradiated with UV light three times at 0.72 J/cm^2^ using a CL-1000 Crosslinker (UVP).

### Expression plasmids

Mammalian expression plasmids for HA-IE1 (pDJK170) and HA-IE2 (pDJK171) were cloned using the pSG5 vector and plasmid for myc-ISG15 (pOK20) was cloned using the pCS3-MT (with a hexa-myc tag) vector using Gateway technology as previously described [59]. Plasmids for myc-ISG15_GG_ (pYJ12), an active form of ISG15 with a termination codon added immediately after the double glycine residues, or myc-ISG15_AA_ (pYJ14), a conjugation-defective mutant in which the double glycine residues are replaced with alanine residues, were produced using the Stratagene QuickChange site-directed mutagenesis protocol. The pSG5-driven plasmids expressing Flag-UbcH8 (pYJ23) and HA-Herc5 (pYJ29) were produced using Gateway technology. Plasmids for SRT-UL26-p21 (pSE124) and Myc-UL26-p21 (pSE107) were cloned using the pcDNA6 (Life Technologies) vector and pCS3-MT vector, respectively, using Gateway technology. For the SRT-UL26-p21 expression plasmid used in ISGylation assays, two lysine residues on the linker between the SRT tag and the UL26 ORF were changed to alanines to block their possible ISGylation, resulting in pYJ178. Site-directed mutagenesis was performed on the pYJ178 background to produce plasmids expressing the lysine to arginine mutant versions of SRT-UL26-p21; K54R (pYJ179), K136R (pYJ180), K136/169R (pYJ182), and K54/136/169R (pYJ183). Plasmids for HA-hUBE1L (pCAGGS-HA-hUBE1L) and Flag-hUbcH8 (pFlagCMV2-UbcH8) and plasmids for S-tagged Herc5 (pCI-neo-S2-Herc5) were kindly provided by Dong-Er Zhang (Moores Cancer Center, University of California, San Diego, La Jolla, CA 92093, USA).

### Yeast two-hybrid assays

Yeast AH109 (*MATa*) cells were transformed with plasmid expressing the GAL4-DNA-binding (DB)-ISG15 (TRP^+^) or GAL4-DB-UBE1L (TRP^+^) fusion protein. Y187 (*MATα*) cells were transformed with plasmid expressing the GAL4-activation domain (A)-HCMV ORF (LEU2^+^) fusion proteins. Each transformant was selected on plates lacking tryptophan (SC-Trp) or leucine (SC-Leu). Trp^+^ and Leu^+^ transformants were mated with each other on YPD plates. Diploid cells (*a/α*) were selected on plates lacking both tryptophan and leucine (SC-TrpLeu). Trp^+^Leu^+^ colonies were tested for their growth on plates that lack tryptophan, leucine and histidine (SC-TrpLeuHis). Cells expressing bait and prey that interact with each other grow on SC-TrpLeuHis. Cells expressing both GAL4- DB-ISG15 and GAL4-A-UBE1L were used as a positive control, whereas cells expressing GAL4-DB-ISG15 and GAL4-A only were used as a negative control.

### Production of the recombinant virus expressing UL26-HA proteins

The Toledo-BAC clone encoding UL26 proteins with a C-terminal HA tag was produced by using a counter-selection BAC modification kit (Gene Bridges). The scheme for bacmid mutagenesis is described in S5 Fig and the LMV primers used for mutagenesis are listed in S1 Table.

### Retroviral vectors

Retroviral vectors expressing IE1 (pYH38) or UL26 (pYJ104), UL26(K136/169R) (pYJ176), and UL26(K54/136/169R) (pYJ177) were produced on the background of pMIN (murine leukemia virus-based retroviral vector) using Gateway technology as previously described [59]. Retroviral vectors expressing shRNA for UbcH8 (pMSCVpuro-shUbcH8) was previously described [5]. To produce retroviral vectors expressing shRNA for UBE1L (pMSCVpuro-shUBE1L-1), short hairpin RNA (shRNA) for UBE1L was amplified with U6 promoter by PCR with primers 5′- TTTGGATCCCAAGGTCGGGCAGGAAGAGGGCCTATTTCC-3′ and 5′-TTTGAATTCAAAAAGGATGATGACAGCAACTTCTCTCTTGAAGAAGTTGCTGTCATCATCCGGTGTTTCGTCCTTTCCACAAGATATATAA-3′ (target sequence underlined). The PCR product was digested with BamHI and EcoRI and ligated to MSCV-PGKpuro (BD Biosciences Clontech) digested with BglII and EcoRI. Recombinant retroviruses were prepared in 293T cells after co-transfection with retroviral vectors together with the packaging plasmids pHIT60 (Gag-Pol) and pMD-G expressing the envelope G protein of vesicular stomatitis virus (VSV) [59] using Metafectene reagents (Biotex). Viral supernatants were collected at 48 h after transfection. HF cells were transduced by retroviruses in the presence of polybrene (7.5 μg/ml). Cells were selected with G418 (0.5 mg/ml) (Calbiochem) and maintained in a medium containing G418 (0.1 mg/ml).

### Lentiviral vectors

Lentiviral vector pLKO.1-TRC control expressing a non-hairpin control RNA (shC) was purchased from Addgene. pLKO.1-based lentiviral vectors expressing shRNA for UBP43 (shUBP43-1: TRCN0000004194 and shUBP43-2: TRCN0000004195) and Herc5 (shHerc5-1: TRCN0000004171 and shHerc5-2: TRCN0000004169) were purchased from Open Biosystems. To produce lentiviruses, 293T cells were transfected with lentiviral vectors together with plasmids pCMV-DR8.91 expressing the gag-pol, tat, and rev proteins of human immunodeficiency virus (HIV) and pMD-G. At 48 h, the viral supernatants were collected and used to transduce HF cells in the presence of polybrene (7.5 μg/ml). The transduced cells were selected with puromycin (1 μg/ml) and maintained in a medium containing puromycin (0.5 μg/ml).

### Antibodies

Mouse monoclonal antibody (MAb) 810R, which detects epitopes present in both IE1 and IE2, was purchased from Chemicon. Mouse MAbs against UL44 (p52) and UL99 (pp28) were obtained from Virusys. Anti-β-actin and anti-α-tubulin mouse MAbs were purchased from Sigma. Anti-HA rat MAb 3F10 and anti-myc mouse MAb 9E10, conjugated with peroxidase or labeled with fluorescein isothiocyanate (FITC), were purchased from Roche. Anti-ISG15 (F-9) and anti-STAT2 mouse MAbs were obtained from Santa Cruz. Mouse MAb against SRT epitope was previously described [91]. UBP43 antibody was previously described [92]. Rabbit polyclonal Ab (PAb) for STAT2 (C-20) and STAT2 phosphorylated at Tyr689 were purchased from Santa Cruz and Upstate, respectively. Rabbit PAb for ISG15 was kindly provided by Chin Ha Chung (Seoul National University, Seoul, Republic of Korea).

### Immunoblot analysis and indirect immunofluorescence assay (IFA)

For immunoblot analysis, cells were washed with phosphate-buffered saline (PBS) and total cell lysates were prepared by boiling the cell pellets in sodium dodecyl sulfate (SDS) loading buffer. Equal amounts of the clarified cell extracts were separated on a SDS-polyacrylamide gel or Gradi-Gel II (Elpis biotech, Republic of Korea) and electroblotted onto nitrocellulose membranes. The blots were blocked by incubation for 30 min at room temperature with PBS plus 0.1% Tween 20 (PBST) containing 5% nonfat dry milk. After being washed with PBST three times, the blots were incubated with the appropriate antibodies in PBST for 1 h at room temperature. After three 5 min washes with PBST, the blots were incubated with horseradish peroxidase-conjugated goat anti-mouse IgG or anti-rabbit IgG (Amersham) for 1 h at room temperature. The blots were then washed three times with PBST, and the protein bands were visualized with enhanced chemiluminescence system (Amersham).

For IFA, cells were fixed in ice-cold methanol for 5 min and rehydrated in cold PBS. Then, the cells were incubated with appropriate primary antibodies in PBS at 37°C for 1 h, followed by incubation with appropriate secondary antibodies at 37°C for 1 h. The mounting solution containing Hoechst and anti-fade reagent (Molecular Probes) was used. For double-labeling, two different antibodies were incubated together. Slides were examined and with a Carl Zeiss LSM710Meta confocal microscope system.

### Luciferase reporter assay

HF cells (2 × 10^5^) were collected and incubated with 200 μl of lysis buffer [40 mM Tris-HCl (pH 7.8), 50 mM NaCl, 2 mM EDTA, 1 mM MgSO_4_, and 1% Triton X-100 plus 5 mM dithiothreitol] for 20 min on ice. The extracts were clarified in a microcentrifuge and 20 μl of extracts were incubated with 350 μl of reaction buffer A (25 mM Gly-Gly pH 7.8, 15 mM ATP and 4 mM EGTA) and then mixed with 100 μl of 1 mM luciferin (Sigma). A TD-20/20 luminometer (Turner Designs) was used for the 10-s assay of the photons produced (measured in relative light units).

### Infectious center assay

The diluted samples were used to inoculate a monolayer of 4 × 10^4^ HF cells in a 24-well plate. At 24 h post infection, cells were fixed with 500 μl of cold methanol for 10 min. The cells were then washed three times in phosphate-buffered saline (PBS) and incubated with anti-IE1 rabbit polyclonal antibody in PBS at 37°C for 1 h, followed by incubation with phosphatase-conjugated anti-rabbit immunoglobulin G (IgG) antibody in phosphate-buffered saline (PBS) at 37°C for 1 h. Finally, the cells were gently washed in PBS and treated with 200 μl of developing solution (nitroblue tetrazolium/5-bromo-4-chloro-3-indolylphosphate) at room temperature for 1 h. The positively stained cells were counted for at least three to five separate fields per well under a light microscope (× 200 magnification).

### Co-immunoprecipitation (co-IP) assays

Co-transfected 293T cells (8 × 10^5^) or virus-infected HF cells were harvested and sonicated in 0.7 ml co-IP buffer [50 mM Tris-Cl (pH 7.4), 50 mM NaF, 5 mM sodium phosphate, 0.1% Triton X-100, containing protease inhibitors (Sigma)] by a microtip probe (Vibra-Cell; Sonics and Materials, Inc., USA) for 10 s (pulse on: l s, pulse off: 3 s). Cell lysates were incubated with appropriate antibodies. After incubation for 16 h at 4°C, 30 μl of a 50% slurry of protein A- and G-Sepharose (Amersham) were added and then the mixture was incubated for 2 h at 4°C to allow adsorption. The mixture was then pelleted and washed 7 times with co-IP buffer. The beads were resuspended and boiled for 5 min in loading buffer. Each sample was analyzed by SDS-PAGE and immunoblotting with appropriate antibodies.

### Co-IP assays to detect ISGylated protein

For co-transfection/ISGylation assays, 293T cells were co-transfected with plasmids expressing target protein and ISGylation enzymes. Co-transfected or virus-infected cells were treated with 0.5 mM NEM (N-ethylmaleimide) for 30 min before they were harvested. Cell pellets were resuspended with 10% SDS lysis buffer containing protease inhibitors and boiled for 10 min. Cell lysates were diluted 10-fold with co-IP buffer (50 Mm Tris-Cl [pH 7.4], 50 mM NaF, 5 mM sodium pyrophosphate, containing protease inhibitors) and sonicated by using a Microtip probe (Vibra cell; Sonics and Materials, Inc.). The clarified cell lysates were incubated with appropriate antibody for 16 h and then with 30 μl of a 50% slurry of protein G for 2 h. The mixture was pelleted and washed seven times with co-IP buffer. The bound proteins were boiled and analyzed by SDS-PAGE followed by immunoblot assays

### Reverse transcription-PCR (RT-PCR) and quantitative real-time RT-PCR (qRT-PCR)

Total RNAs were isolated from 2 × 10^5^ cells using TRIzol reagent (Invitrogen) and MaXtract High Density (Qiagen). First-strand cDNA was synthesized by using the random hexamer primers in the SuperScript III system (Invitrogen). Quantitative real-time TR-PCR (qRT-PCR) was performed using the Applied Biosystems ABI Prism SDS software and the following primers: for ISG15, 5ʹ-GCTGGGACCTGACGGTG-3ʹ (sense) and 5ʹ-TTAGCTCCGCCCGCCAG-3ʹ (anti-sense); for UBE1L, 5ʹ-AGGTGGCCAAGAACTTGGTT-3ʹ (sense) and 5ʹ-CACCACCTGGAAGTCCAACA-3ʹ (anti-sense); for UbcH8, 5ʹ-AACCTGTCCAGCGATGATGC-3ʹ (sense) and 5ʹ-TGGTGCAAGGCTTCCAGTTC-3ʹ (anti-sense); for Herc5, 5ʹ-GGGATGAAAGTGCTGAGGAG-3ʹ (sense) and 5ʹ-CATTTTCTGAAGCGTCCACA-3ʹ (anti-sense); for β-actin, 5ʹ-AGCGGGAAATCGTGCGTG-3ʹ (sense) and 5ʹ-CAGGGTACATGGTGGTGCC-3ʹ (anti-sense).

### Statistical analysis

Statistical significances were determined using the Student’s *t*-test and are indicated by *P<0.05, **P<0.01, or ***P<0.001.

## Supporting Information

S1 Fig Effects of UBE1L and UbcH8-knockdown on HCMV growth. (A) 293T cells were co-transfected with MSCV retroviral vectors expressing shRNA for UBE1L (shUBE1L-1 or shUBE1L-2) and plasmids encoding HA-UBE1L or HA-AML1b as indicated. At 48 h after transfection, cell lysates were immunoblotted with anti-HA antibody. The results showed that shUBE1L-1 specifically reduced UBE1L expression. (B) HeLa cells transduced by MSCV or shUBE1L-1 (hereafter shUBE1L)-expressing MSCV were treated or not with IFNxα (5,000 U/ml) for 24 h. Cell lysates were immunoblotted with antibodies for ISG15 and tubulin-a. (C) Control HF cells or cells expressing UbcH8-specific shRNA were mock-infected or infected with UV-HCMV. The levels of UbcH8 were determined by immunoblotting with anti-UbcH8 rabbit polyclonal antibodies. (D) Control HF cells or cells expressing UBE1L- or UbcH8-specific shRNA were mock-infected or infected with UV-HCMV. The levels of UBE1L and UbcH8 transcripts were determined at 24 h after infection by qRT-PCR. The β-actin transcript levels were used for normalization. Values are the averages of duplicated assays; error ranges are indicated. (E) HF-shRNA cells were infected or not with UV-HCMV at an MOI of 3. At 24 h after infection, immunoblotting was performed with antibodies for ISG15 and β-actin. (F) HF-shRNA cells were infected with the recombinant virus containing the GFP expression cassette (HCMV-GFP) at an MOI of 0.1. GFP images of cells were taken at 7 days after infection. (G) HF-shRNA cells were infected with HCMV at an MOI of 0.1. At 9 days after infection, the viral supernatants were collected and the levels of progeny virions were measured by infectious center assays. Statistical significances were determined using the Student’s *t*-test and are indicated by **P<0.01.
(TIF)

S2 Fig Effect of ectopic expression of ISG15 and ISGylation enzymes on HCMV growth. HF cells (2 × 10^5^) were transfected by electroporation with plasmids expressing myc-ISG15_GG_ (or myc-ISG15_AA_), HA-UBE1L, flag-UbcH8, and HA-Herc5 in combinations as indicated. At 24 h after electroporation, cells were infected with HCMV at an MOI of 1. At 6 days after infection, viral titers in the culture supernatants were measured by infectious center assays (top). The results were shown as averages in two experiments. The levels of exogenously expressed myc-ISG15 proteins, myc-ISG15 conjugates, and β-actin were shown by immunoblotting (bottom).
(TIF)

S3 Fig Effect of ISGylation on the activation of HCMV promoters. (A-B) HF cells were co-transfected with reporter plasmids containing MIE-Luc, UL112-113-Luc, Pol-Luc, or pp28-Luc reporter gene and effector plasmids as indicated. At 48 after transfection, cell lysates were prepared and assayed for the luciferase activity. The results shown are the mean values for the three independent experiments with standard errors. The expression levels of myc-ISG15 and myc-ISG15 conjugates, HA-IE2, and β-actin in transfected cells were shown by immunoblotting. Statistical significances were determined using the Student’s *t*-test and are indicated by *P<0.05 or **P<0.01.
(TIF)

S4 Fig Interactions of HCMV proteins with ISG15 and UBE1L in co-IP assays. (A) 293T cells were co-transfected with plasmids encoding GFP-ISG15_AA_ or myc-ORFs, as indicated. At 48 h after transfection, cell lysates were prepared and immunoprecipitated with anti-myc antibody, followed by immunoblotting with anti-GFP antibody. To determine the expression levels of each protein, whole cell lysate were also immunoblotted. (B) 293T cells were co-transfected with plasmids encoding HA-UBE1L or myc-ORFs, as indicated. At 48 h after transfection, cell lysates were prepared and immunoprecipitated with anti-myc antibody, followed by immunoblotting with anti-HA antibody.
(TIF)

S5 Fig Production of the recombinant HCMV expressing UL26 proteins with a C-terminal HA tag. (A) The scheme for Toledo-bacmid mutagenesis. The HCMV (Toledo) bacterial artificial chromosome (BAC) clone (Toledo-BAC)[93] was a gift from Hua Zhu (UMDNJ-New Jersey Medical School, Newark, NJ, USA). The rpsL-neo cassettes were PCR-amplified using LMV1766/1767 primers containing homology arms consisting of 50 nucleotides upstream and downstream of the target region plus 24 nucleotides homologous to the rpsL-neo cassette. The amplified rpsL-neo fragments with homology arms were purified and introduced into *E*. *coli* GS243 containing wild-type Toledo-BAC for recombination by electroporation using a Gene Pulser II (Bio-Rad). The intermediate Toledo-BAC constructs containing the rpsL-neo cassette were selected on Luria Broth (LB) plates containing kanamycin. Next, the rpsL-neo cassette was replaced by annealed oligo DNAs (LMV1768/1769) consisting of only homology arms (50 nucleotides upstream and downstream of the target region). The ΔUL26 Toledo-BAC was selected on LB plates containing streptomycin. The mutated regions were amplified by PCR and sequenced to verify the desired mutation. The Toledo-BAC encoding UL26-HA was generated from the mutant Toledo-BAC. First, the rps-neo cassettes flanked by homology arms were inserted again into the mutant Toledo-BAC. Next, DNA fragments containing the wild-type UL26 gene with a HA tag at its C-terminus were PCR amplified by 2-steps using LMV1805/1812 and LMV1805/1772. The amplified UL26-HA gene was then inserted into the Toledo-BAC containing the rps-neo cassette by homologous recombination. The LMV primers used for mutagenesis are listed in S1 Table. (B) The regions containing the UL26 ORF from Wt, ΔUL26, and UL26-HA bacmid DNAs were PCR amplified with LMV1764/1765. (C) Wt, ΔUL26, and UL26-HA bacmid DNAs were digested with BglII and the digestion patterns were compared via agarose gel electrophoresis. The bands corresponding to 5,226 and 5,253 bp from wild-type and UL26-HA bacmids, respectively, and a band of 4,660 bp from ΔUL26 bacmid were indicated as arrowheads.
(TIF)

S6 Fig Specific binding of pUL26 with ISG15, UBE1L, and Hec5 in co-IP assays and broad ISGylation of proteins in cotransfection/ISGylation assays. (A-C) 293T cells were co-transfected with plasmids encoding SRT-ISG15, HA-UBE1L, HA-Herc5, or myc-ORFs, as indicated. At 48 h after transfection, cell lysates were prepared and immunoprecipitated with anti-myc antibody, followed by immunoblotting with anti-SRT antibody (A) or anti-HA antibody (B and C). To determine the expression levels of each protein, whole cell lysate were also immunoblotted. (D) Co-transfection/ISGylation assays. 293T cells were co-transfected with plasmid expressing SRT-tagged ORF (UL26, UL85, and UL71), myc-ISG15 (with GG or AA terminus), HA-UBE1L, Flag-UbcH8, or HA-Herc5 as indicated. At 48 h after transfection, cell lysates were immunoprecipitated with anti-SRT antibody, followed by immunoblotting with anti-myc antibody. Whole cell lysates were immunoblotted with anti-SRT antibody to determine the expression levels of each protein.
(TIF)

S7 Fig Lack of ISGylation and ISGylation inhibitory effect of IE2. Comparative co-transfection/ISGylation assays for UL26 and IE2 were performed in 293T cells with or without increasing amounts of plasmids expressing SRT-UL26-p21 or SRT-IE2 IE1 as in Fig 2D. Cell lysates were prepared and immunoprecipitated with anti-SRT antibody, followed by immunoblotting with anti-myc antibody. Whole cell lysates were immunoblotted with anti-SRT antibody to determine the expression levels of UL26-p21 and IE2, or with anti-myc antibody to determine the effect of UL26-p21 or IE2 expression on ISGylation.
(TIF)

S8 Fig Comparison of ISGylation between wild-type and ΔUL26 virus infected cells. HF cells were mock-infected or infected with wild-type or ΔUL26 mutant virus (Ad169) at an MOI of 0.2. Cell lysates were immunoblotted at the indicated time points with antibodies for ISG15, viral proteins (IE1, IE2, and UL26), and β-actin.
(TIF)

S1 Table PCR primers used for bacmid mutagenesis. (TIF)

S2 Table Summary of the HCMV proteins that interacted with ISG15 and UBE1L in yeast two-hybrid assays and co-IP assays. (TIF)

## References

[1] Sadler AJ, Williams BR. Interferon-inducible antiviral effectors. Nat Rev Immunol. 2008;8(7):559–68. 10.1038/nri2314 18575461 PMC2522268

[2] Jeon YJ, Yoo HM, Chung CH. ISG15 and immune diseases. Biochim Biophys Acta. 2010;1802(5):485–96. 10.1016/j.bbadis.2010.02.006 20153823 PMC7127291

[3] Yuan W, Krug RM. Influenza B virus NS1 protein inhibits conjugation of the interferon (IFN)-induced ubiquitin-like ISG15 protein. Embo J. 2001;20(3):362–71. 11157743 10.1093/emboj/20.3.362 PMC133459

[4] Zhao C, Beaudenon SL, Kelley ML, Waddell MB, Yuan W, Schulman BA, et al The UbcH8 ubiquitin E2 enzyme is also the E2 enzyme for ISG15, an IFN-alpha/beta-induced ubiquitin-like protein. Proc Natl Acad Sci U S A. 2004;101(20):7578–82. 15131269 10.1073/pnas.0402528101 PMC419648

[5] Kim KI, Giannakopoulos NV, Virgin HW, Zhang DE. Interferon-inducible ubiquitin E2, Ubc8, is a conjugating enzyme for protein ISGylation. Mol Cell Biol. 2004;24(21):9592–600. 15485925 10.1128/MCB.24.21.9592-9600.2004 PMC522249

[6] Dastur A, Beaudenon S, Kelley M, Krug RM, Huibregtse JM. Herc5, an interferon-induced HECT E3 enzyme, is required for conjugation of ISG15 in human cells. J Biol Chem. 2006;281(7):4334–8. 16407192 10.1074/jbc.M512830200

[7] Wong JJ, Pung YF, Sze NS, Chin KC. HERC5 is an IFN-induced HECT-type E3 protein ligase that mediates type I IFN-induced ISGylation of protein targets. Proc Natl Acad Sci U S A. 2006;103(28):10735–40. 16815975 10.1073/pnas.0600397103 PMC1484417

[8] Zou W, Zhang DE. The interferon-inducible ubiquitin-protein isopeptide ligase (E3) EFP also functions as an ISG15 E3 ligase. J Biol Chem. 2006;281(7):3989–94. 16352599 10.1074/jbc.M510787200

[9] Okumura F, Zou W, Zhang DE. ISG15 modification of the eIF4E cognate 4EHP enhances cap structure-binding activity of 4EHP. Genes Dev. 2007;21(3):255–60. 17289916 10.1101/gad.1521607 PMC1785121

[10] Haas AL, Ahrens P, Bright PM, Ankel H. Interferon induces a 15-kilodalton protein exhibiting marked homology to ubiquitin. J Biol Chem. 1987;262(23):11315–23. 2440890

[11] Nyman TA, Matikainen S, Sareneva T, Julkunen I, Kalkkinen N. Proteome analysis reveals ubiquitin-conjugating enzymes to be a new family of interferon-alpha-regulated genes. Eur J Biochem. 2000;267(13):4011–9. 10866800 10.1046/j.1432-1327.2000.01433.x

[12] Malakhova O, Malakhov M, Hetherington C, Zhang DE. Lipopolysaccharide activates the expression of ISG15-specific protease UBP43 via interferon regulatory factor 3. J Biol Chem. 2002;277(17):14703–11. 11854279 10.1074/jbc.M111527200

[13] Li XL, Blackford JA, Judge CS, Liu M, Xiao W, Kalvakolanu DV, et al RNase-L-dependent destabilization of interferon-induced mRNAs. A role for the 2-5A system in attenuation of the interferon response. J Biol Chem. 2000;275(12):8880–8. 10722734 10.1074/jbc.275.12.8880

[14] Kang D, Jiang H, Wu Q, Pestka S, Fisher PB. Cloning and characterization of human ubiquitin-processing protease-43 from terminally differentiated human melanoma cells using a rapid subtraction hybridization protocol RaSH. Gene. 2001;267(2):233–42. 11313150 10.1016/s0378-1119(01)00384-5

[15] Malakhova OA, Kim KI, Luo JK, Zou W, Kumar KG, Fuchs SY, et al UBP43 is a novel regulator of interferon signaling independent of its ISG15 isopeptidase activity. EMBO J. 2006;25(11):2358–67. 16710296 10.1038/sj.emboj.7601149 PMC1478183

[16] Shi HX, Yang K, Liu X, Liu XY, Wei B, Shan YF, et al Positive regulation of interferon regulatory factor 3 activation by Herc5 via ISG15 modification. Mol Cell Biol. 2010;30(10):2424–36. 10.1128/MCB.01466-09 20308324 PMC2863703

[17] Arimoto K, Konishi H, Shimotohno K. UbcH8 regulates ubiquitin and ISG15 conjugation to RIG-I. Mol Immunol. 2008;45(4):1078–84. 17719635 10.1016/j.molimm.2007.07.021

[18] Giannakopoulos NV, Luo JK, Papov V, Zou W, Lenschow DJ, Jacobs BS, et al Proteomic identification of proteins conjugated to ISG15 in mouse and human cells. Biochem Biophys Res Commun. 2005;336(2):496–506. 16139798 10.1016/j.bbrc.2005.08.132

[19] Malakhov MP, Kim KI, Malakhova OA, Jacobs BS, Borden EC, Zhang DE. High-throughput immunoblotting. Ubiquitiin-like protein ISG15 modifies key regulators of signal transduction. J Biol Chem. 2003;278(19):16608–13. 12582176 10.1074/jbc.M208435200

[20] Zhao C, Denison C, Huibregtse JM, Gygi S, Krug RM. Human ISG15 conjugation targets both IFN-induced and constitutively expressed proteins functioning in diverse cellular pathways. Proc Natl Acad Sci U S A. 2005;102(29):10200–5. 16009940 10.1073/pnas.0504754102 PMC1177427

[21] Lu G, Reinert JT, Pitha-Rowe I, Okumura A, Kellum M, Knobeloch KP, et al ISG15 enhances the innate antiviral response by inhibition of IRF-3 degradation. Cell Mol Biol (Noisy-le-grand). 2006;52(1):29–41. 16914094

[22] Hsiang TY, Zhao C, Krug RM. Interferon-induced ISG15 conjugation inhibits influenza A virus gene expression and replication in human cells. J Virol. 2009;83(12):5971–7. 10.1128/JVI.01667-08 19357168 PMC2687383

[23] Lenschow DJ, Lai C, Frias-Staheli N, Giannakopoulos NV, Lutz A, Wolff T, et al IFN-stimulated gene 15 functions as a critical antiviral molecule against influenza, herpes, and Sindbis viruses. Proc Natl Acad Sci U S A. 2007;104(4):1371–6. 17227866 10.1073/pnas.0607038104 PMC1783119

[24] Tang Y, Zhong G, Zhu L, Liu X, Shan Y, Feng H, et al Herc5 Attenuates Influenza A Virus by Catalyzing ISGylation of Viral NS1 Protein. J Immunol. 2010;184(10):5777–90. 10.4049/jimmunol.0903588 20385878

[25] Lai C, Struckhoff JJ, Schneider J, Martinez-Sobrido L, Wolff T, Garcia-Sastre A, et al Mice lacking the ISG15 E1 enzyme UbE1L demonstrate increased susceptibility to both mouse-adapted and non-mouse-adapted influenza B virus infection. J Virol. 2009;83(2):1147–51. 10.1128/JVI.00105-08 19004958 PMC2612374

[26] Okumura A, Lu G, Pitha-Rowe I, Pitha PM. Innate antiviral response targets HIV-1 release by the induction of ubiquitin-like protein ISG15. Proc Natl Acad Sci U S A. 2006;103(5):1440–5. 16434471 10.1073/pnas.0510518103 PMC1360585

[27] Kunzi MS, Pitha PM. Role of interferon-stimulated gene ISG-15 in the interferon-omega-mediated inhibition of human immunodeficiency virus replication. J Interferon Cytokine Res. 1996;16(11):919–27. 8938567 10.1089/jir.1996.16.919

[28] Chua PK, McCown MF, Rajyaguru S, Kular S, Varma R, Symons J, et al Modulation of alpha interferon anti-hepatitis C virus activity by ISG15. J Gen Virol. 2009;90(Pt 12):2929–39. 10.1099/vir.0.013128-0 19656964

[29] Kim MJ, Yoo JY. Inhibition of hepatitis C virus replication by IFN-mediated ISGylation of HCV-NS5A. J Immunol. 2010;185(7):4311–8. 10.4049/jimmunol.1000098 20810994

[30] Domingues P, Bamford CG, Boutell C, McLauchlan J. Inhibition of Hepatitis C Virus RNA Replication by ISG15 does not Require Its Conjugation to Protein Substrates by the HERC5 E3 Ligase. J Gen Virol. 2015. 10.1099/jgv.0.000283 PMC4806579 26361997

[31] Hsiao NW, Chen JW, Yang TC, Orloff GM, Wu YY, Lai CH, et al ISG15 over-expression inhibits replication of the Japanese encephalitis virus in human medulloblastoma cells. Antiviral Res. 2010;85(3):504–11. 10.1016/j.antiviral.2009.12.007 20035788

[32] Giannakopoulos NV, Arutyunova E, Lai C, Lenschow DJ, Haas AL, Virgin HW. ISG15 Arg151 and the ISG15-conjugating enzyme UbE1L are important for innate immune control of Sindbis virus. J Virol. 2009;83(4):1602–10. 10.1128/JVI.01590-08 19073728 PMC2643764

[33] Lenschow DJ, Giannakopoulos NV, Gunn LJ, Johnston C, O'Guin AK, Schmidt RE, et al Identification of interferon-stimulated gene 15 as an antiviral molecule during Sindbis virus infection in vivo. J Virol. 2005;79(22):13974–83. 16254333 10.1128/JVI.79.22.13974-13983.2005 PMC1280211

[34] Malakhova OA, Zhang DE. ISG15 inhibits Nedd4 ubiquitin E3 activity and enhances the innate antiviral response. J Biol Chem. 2008;283(14):8783–7. 10.1074/jbc.C800030200 18287095 PMC2276364

[35] Okumura A, Pitha PM, Harty RN. ISG15 inhibits Ebola VP40 VLP budding in an L-domain-dependent manner by blocking Nedd4 ligase activity. Proc Natl Acad Sci U S A. 2008;105(10):3974–9. 10.1073/pnas.0710629105 18305167 PMC2268823

[36] Guerra S, Caceres A, Knobeloch KP, Horak I, Esteban M. Vaccinia virus E3 protein prevents the antiviral action of ISG15. PLoS Pathog. 2008;4(7):e1000096 10.1371/journal.ppat.1000096 18604270 PMC2434199

[37] Dai J, Pan W, Wang P. ISG15 facilitates cellular antiviral response to dengue and west nile virus infection in vitro. Virol J. 2011;8:468 10.1186/1743-422X-8-468 21992229 PMC3215395

[38] Sun Z, Li Y, Ransburgh R, Snijder EJ, Fang Y. Nonstructural protein 2 of porcine reproductive and respiratory syndrome virus inhibits the antiviral function of interferon-stimulated gene 15. J Virol. 2012;86(7):3839–50. 10.1128/JVI.06466-11 22258253 PMC3302520

[39] Jacobs SR, Stopford CM, West JA, Bennett CL, Giffin L, Damania B. Kaposi's Sarcoma-Associated Herpesvirus Viral Interferon Regulatory Factor 1 Interacts with a Member of the Interferon-Stimulated Gene 15 Pathway. J Virol. 2015;89(22):11572–83. 10.1128/JVI.01482-15 26355087 PMC4645652

[40] Gonzalez-Sanz R, Mata M, Bermejo-Martin J, Alvarez A, Cortijo J, Melero JA, et al ISG15 is upregulated in respiratory syncytial virus infection and reduces virus growth through protein ISGylation. J Virol. 2016;90(7):3428–38. 10.1128/JVI.02695-15 26763998 PMC4794669

[41] Durfee LA, Lyon N, Seo K, Huibregtse JM. The ISG15 conjugation system broadly targets newly synthesized proteins: implications for the antiviral function of ISG15. Mol Cell. 2010;38(5):722–32. 10.1016/j.molcel.2010.05.002 20542004 PMC2887317

[42] Zhao C, Hsiang TY, Kuo RL, Krug RM. ISG15 conjugation system targets the viral NS1 protein in influenza A virus-infected cells. Proc Natl Acad Sci U S A. 2010;107(5):2253–8. 10.1073/pnas.0909144107 20133869 PMC2836655

[43] Seo EJ, Leis J. Budding of Enveloped Viruses: Interferon-Induced ISG15-Antivirus Mechanisms Targeting the Release Process. Adv Virol. 2012;2012:532723 10.1155/2012/532723 22666250 PMC3362814

[44] Werneke SW, Schilte C, Rohatgi A, Monte KJ, Michault A, Arenzana-Seisdedos F, et al ISG15 is critical in the control of Chikungunya virus infection independent of UbE1L mediated conjugation. PLoS Pathog. 2011;7(10):e1002322 10.1371/journal.ppat.1002322 22028657 PMC3197620

[45] Kim MJ, Hwang SY, Imaizumi T, Yoo JY. Negative feedback regulation of RIG-I-mediated antiviral signaling by interferon-induced ISG15 conjugation. J Virol. 2008;82(3):1474–83. 18057259 10.1128/JVI.01650-07 PMC2224411

[46] Chen L, Sun J, Meng L, Heathcote J, Edwards AM, McGilvray ID. ISG15, a ubiquitin-like interferon-stimulated gene, promotes hepatitis C virus production in vitro: implications for chronic infection and response to treatment. J Gen Virol. 2010;91(Pt 2):382–8. 10.1099/vir.0.015388-0 19846672

[47] Broering R, Zhang X, Kottilil S, Trippler M, Jiang M, Lu M, et al The interferon stimulated gene 15 functions as a proviral factor for the hepatitis C virus and as a regulator of the IFN response. Gut. 2010;59(8):1111–9. 10.1136/gut.2009.195545 20639253

[48] Bogunovic D, Boisson-Dupuis S, Casanova JL. ISG15: leading a double life as a secreted molecule. Exp Mol Med. 2013;45:e18 10.1038/emm.2013.36 23579383 PMC3641400

[49] Campbell JA, Lenschow DJ. Emerging roles for immunomodulatory functions of free ISG15. J Interferon Cytokine Res. 2013;33(12):728–38. 10.1089/jir.2013.0064 24010825 PMC3868375

[50] Bogunovic D, Byun M, Durfee LA, Abhyankar A, Sanal O, Mansouri D, et al Mycobacterial disease and impaired IFN-gamma immunity in humans with inherited ISG15 deficiency. Science. 2012;337(6102):1684–8. 22859821 10.1126/science.1224026 PMC3507439

[51] Pohl C, Dikic I. Fighting mycobacteria through ISGylation. EMBO Rep. 2012;13(10):872–3. 10.1038/embor.2012.129 22940737 PMC3464402

[52] Zhang X, Bogunovic D, Payelle-Brogard B, Francois-Newton V, Speer SD, Yuan C, et al Human intracellular ISG15 prevents interferon-alpha/beta over-amplification and auto-inflammation. Nature. 2015;517(7532):89–93. 10.1038/nature13801 25307056 PMC4303590

[53] Speer SD, Li Z, Buta S, Payelle-Brogard B, Qian L, Vigant F, et al ISG15 deficiency and increased viral resistance in humans but not mice. Nature communications. 2016;7:11496 10.1038/ncomms11496 27193971 PMC4873964

[54] Mocarski ES, Shenk T, Griffiths PD, Pass RF. Cytomegaloviruses, p. 1960–2014. In Knipe D. M., Howley P. M., Cohen J. I., Griffin D. E., Lamb R. A., Martin M. A., Racaniello V. R., and Roizman B. (ed.), Fields virology. Philadelphia, PA: Lippincott Williams & Wilkins; 2013.

[55] Browne EP, Shenk T. Human cytomegalovirus UL83-coded pp65 virion protein inhibits antiviral gene expression in infected cells. Proc Natl Acad Sci U S A. 2003;100(20):11439–44. 12972646 10.1073/pnas.1534570100 PMC208776

[56] Taylor RT, Bresnahan WA. Human cytomegalovirus immediate-early 2 gene expression blocks virus-induced beta interferon production. J Virol. 2005;79(6):3873–7. 15731283 10.1128/JVI.79.6.3873-3877.2005 PMC1075717

[57] Abate DA, Watanabe S, Mocarski ES. Major human cytomegalovirus structural protein pp65 (ppUL83) prevents interferon response factor 3 activation in the interferon response. J Virol. 2004;78(20):10995–1006. 15452220 10.1128/JVI.78.20.10995-11006.2004 PMC521853

[58] Paulus C, Krauss S, Nevels M. A human cytomegalovirus antagonist of type I IFN-dependent signal transducer and activator of transcription signaling. Proc Natl Acad Sci U S A. 2006;103(10):3840–5. 16497831 10.1073/pnas.0600007103 PMC1533784

[59] Huh YH, Kim YE, Kim ET, Park JJ, Song MJ, Zhu H, et al Binding STAT2 by the acidic domain of human cytomegalovirus IE1 promotes viral growth and is negatively regulated by SUMO. J Virol. 2008;82(21):10444–54. 10.1128/JVI.00833-08 18701593 PMC2573188

[60] Krauss S, Kaps J, Czech N, Paulus C, Nevels M. Physical requirements and functional consequences of complex formation between the cytomegalovirus IE1 protein and human STAT2. J Virol. 2009;83(24):12854–70. 10.1128/JVI.01164-09 19812155 PMC2786848

[61] Kim YE, Ahn JH. Positive role of promyelocytic leukemia protein in type I interferon response and its regulation by human cytomegalovirus. PLoS Pathog. 2015;11(3):e1004785 10.1371/journal.ppat.1004785 25812002 PMC4374831

[62] Scherer M, Otto V, Stump JD, Klingl S, Muller R, Reuter N, et al Characterization of Recombinant Human Cytomegaloviruses Encoding IE1 Mutants L174P and 1–382 Reveals that Viral Targeting of PML Bodies Perturbs both Intrinsic and Innate Immune Responses. J Virol. 2015;90(3):1190–205. 10.1128/JVI.01973-15 26559840 PMC4719593

[63] Miller DM, Rahill BM, Boss JM, Lairmore MD, Durbin JE, Waldman JW, et al Human cytomegalovirus inhibits major histocompatibility complex class II expression by disruption of the Jak/Stat pathway. J Exp Med. 1998;187(5):675–83. 9480977 10.1084/jem.187.5.675 PMC2212176

[64] Miller DM, Zhang Y, Rahill BM, Waldman WJ, Sedmak DD. Human cytomegalovirus inhibits IFN-alpha-stimulated antiviral and immunoregulatory responses by blocking multiple levels of IFN-alpha signal transduction. J Immunol. 1999;162(10):6107–13. 10229853

[65] Le VT, Trilling M, Wilborn M, Hengel H, Zimmermann A. Human cytomegalovirus interferes with signal transducer and activator of transcription (STAT) 2 protein stability and tyrosine phosphorylation. J Gen Virol. 2008;89(Pt 10):2416–26. 10.1099/vir.0.2008/001669-0 18796709

[66] Knoblach T, Grandel B, Seiler J, Nevels M, Paulus C. Human cytomegalovirus IE1 protein elicits a type II interferon-like host cell response that depends on activated STAT1 but not interferon-gamma. PLoS Pathog. 2011;7(4):e1002016 10.1371/journal.ppat.1002016 21533215 PMC3077363

[67] Greaves RF, Mocarski ES. Defective growth correlates with reduced accumulation of a viral DNA replication protein after low-multiplicity infection by a human cytomegalovirus ie1 mutant. J Virol. 1998;72(1):366–79. 9420235 10.1128/jvi.72.1.366-379.1998 PMC109384

[68] Loeb KR, Haas AL. The interferon-inducible 15-kDa ubiquitin homolog conjugates to intracellular proteins. J Biol Chem. 1992;267(11):7806–13. 1373138

[69] Malakhova OA, Yan M, Malakhov MP, Yuan Y, Ritchie KJ, Kim KI, et al Protein ISGylation modulates the JAK-STAT signaling pathway. Genes Dev. 2003;17(4):455–60. 12600939 10.1101/gad.1056303 PMC195994

[70] Kim ET, Kim YE, Kim YJ, Lee MK, Hayward GS, Ahn JH. Analysis of Human Cytomegalovirus-Encoded SUMO Targets and Temporal Regulation of SUMOylation of the Immediate-Early Proteins IE1 and IE2 during Infection. PLoS One. 2014;9(7):e103308 10.1371/journal.pone.0103308 25050850 PMC4106884

[71] Stamminger T, Gstaiger M, Weinzierl K, Lorz K, Winkler M, Schaffner W. Open reading frame UL26 of human cytomegalovirus encodes a novel tegument protein that contains a strong transcriptional activation domain. J Virol. 2002;76(10):4836–47. 11967300 10.1128/JVI.76.10.4836-4847.2002 PMC136153

[72] Lorz K, Hofmann H, Berndt A, Tavalai N, Mueller R, Schlotzer-Schrehardt U, et al Deletion of open reading frame UL26 from the human cytomegalovirus genome results in reduced viral growth, which involves impaired stability of viral particles. J Virol. 2006;80(11):5423–34. 16699023 10.1128/JVI.02585-05 PMC1472153

[73] Mathers C, Spencer CM, Munger J. Distinct domains within the human cytomegalovirus U(L)26 protein are important for wildtype viral replication and virion stability. PLoS One. 2014;9(2):e88101 10.1371/journal.pone.0088101 24505393 PMC3914908

[74] Mathers C, Schafer X, Martinez-Sobrido L, Munger J. The human cytomegalovirus UL26 protein antagonizes NF-kappaB activation. J Virol. 2014;88(24):14289–300. 10.1128/JVI.02552-14 25275128 PMC4249132

[75] Fan JB, Arimoto K, Motamedchaboki K, Yan M, Wolf DA, Zhang DE. Identification and characterization of a novel ISG15-ubiquitin mixed chain and its role in regulating protein homeostasis. Scientific reports. 2015;5:12704 10.1038/srep12704 26226047 PMC4520236

[76] Zou W, Wang J, Zhang DE. Negative regulation of ISG15 E3 ligase EFP through its autoISGylation. Biochem Biophys Res Commun. 2007;354(1):321–7. 17222803 10.1016/j.bbrc.2006.12.210 PMC1858649

[77] Kim ET, Kim YE, Huh YH, Ahn JH. Role of noncovalent SUMO binding by the human cytomegalovirus IE2 transactivator in lytic growth. J Virol. 2010;84(16):8111–23. 10.1128/JVI.00459-10 20519406 PMC2916544

[78] Ferreira HC, Luke B, Schober H, Kalck V, Lingner J, Gasser SM. The PIAS homologue Siz2 regulates perinuclear telomere position and telomerase activity in budding yeast. Nat Cell Biol. 2011;13(7):867–74. 10.1038/ncb2263 21666682

[79] Hang LE, Liu X, Cheung I, Yang Y, Zhao X. SUMOylation regulates telomere length homeostasis by targeting Cdc13. Nature structural & molecular biology. 2011;18(8):920–6. 10.1038/nsmb.2100 PMC3291484 21743457

[80] Wu CS, Ouyang J, Mori E, Nguyen HD, Marechal A, Hallet A, et al SUMOylation of ATRIP potentiates DNA damage signaling by boosting multiple protein interactions in the ATR pathway. Genes & development. 2014;28(13):1472–84. 24990965 10.1101/gad.238535.114 PMC4083090

[81] Garg M, Gurung RL, Mansoubi S, Ahmed JO, Dave A, Watts FZ, et al Tpz1TPP1 SUMOylation reveals evolutionary conservation of SUMO-dependent Stn1 telomere association. EMBO Rep. 2014;15(8):871–7. 10.15252/embr.201438919 24925530 PMC4197044

[82] Chang YG, Yan XZ, Xie YY, Gao XC, Song AX, Zhang DE, et al Different roles for two ubiquitin-like domains of ISG15 in protein modification. J Biol Chem. 2008;283(19):13370–7. 10.1074/jbc.M800162200 18356159

[83] Tandon R, AuCoin DP, Mocarski ES. Human cytomegalovirus exploits ESCRT machinery in the process of virion maturation. J Virol. 2009;83(20):10797–807. 10.1128/JVI.01093-09 19640981 PMC2753131

[84] Tandon R, Mocarski ES. Viral and host control of cytomegalovirus maturation. Trends Microbiol. 2012;20(8):392–401. 10.1016/j.tim.2012.04.008 22633075 PMC3408842

[85] Calistri A, Sette P, Salata C, Cancellotti E, Forghieri C, Comin A, et al Intracellular trafficking and maturation of herpes simplex virus type 1 gB and virus egress require functional biogenesis of multivesicular bodies. J Virol. 2007;81(20):11468–78. 17686835 10.1128/JVI.01364-07 PMC2045546

[86] Crump CM, Yates C, Minson T. Herpes simplex virus type 1 cytoplasmic envelopment requires functional Vps4. J Virol. 2007;81(14):7380–7. 17507493 10.1128/JVI.00222-07 PMC1933334

[87] Frias-Staheli N, Giannakopoulos NV, Kikkert M, Taylor SL, Bridgen A, Paragas J, et al Ovarian tumor domain-containing viral proteases evade ubiquitin- and ISG15-dependent innate immune responses. Cell Host Microbe. 2007;2(6):404–16. 18078692 10.1016/j.chom.2007.09.014 PMC2184509

[88] Lindner HA, Lytvyn V, Qi H, Lachance P, Ziomek E, Menard R. Selectivity in ISG15 and ubiquitin recognition by the SARS coronavirus papain-like protease. Arch Biochem Biophys. 2007;466(1):8–14. 17692280 10.1016/j.abb.2007.07.006 PMC7094341

[89] Munger J, Yu D, Shenk T. UL26-deficient human cytomegalovirus produces virions with hypophosphorylated pp28 tegument protein that is unstable within newly infected cells. J Virol. 2006;80(7):3541–8. 16537622 10.1128/JVI.80.7.3541-3548.2006 PMC1440364

[90] Kim ET, Oh SE, Lee YO, Gibson W, Ahn JH. Cleavage specificity of the UL48 deubiquitinating protease activity of human cytomegalovirus and the growth of an active-site mutant virus in cultured cells. J Virol. 2009;83(23):12046–56. 10.1128/JVI.00411-09 19759126 PMC2786740

[91] Lee JM, Kang HJ, Lee HR, Choi CY, Jang WJ, Ahn JH. PIAS1 enhances SUMO-1 modification and the transactivation activity of the major immediate-early IE2 protein of human cytomegalovirus. FEBS Lett. 2003;555(2):322–8. 14644436 10.1016/s0014-5793(03)01268-7

[92] Malakhov MP, Malakhova OA, Kim KI, Ritchie KJ, Zhang DE. UBP43 (USP18) specifically removes ISG15 from conjugated proteins. J Biol Chem. 2002;277(12):9976–81. 11788588 10.1074/jbc.M109078200

[93] Murphy E, Yu D, Grimwood J, Schmutz J, Dickson M, Jarvis MA, et al Coding potential of laboratory and clinical strains of human cytomegalovirus. Proc Natl Acad Sci U S A 2003;100(25):14976–81. 14657367 10.1073/pnas.2136652100 PMC299866

