# Supplementary figures and images for: Consecutive Inhibition of ISG15 Expression and ISGylation by Cytomegalovirus Regulators
Source: PLoS Pathog. 2016 Aug 26;12(8):e1005850. doi: 10.1371/journal.ppat.1005850 (PMC5001722; doi:10.1371/journal.ppat.1005850)

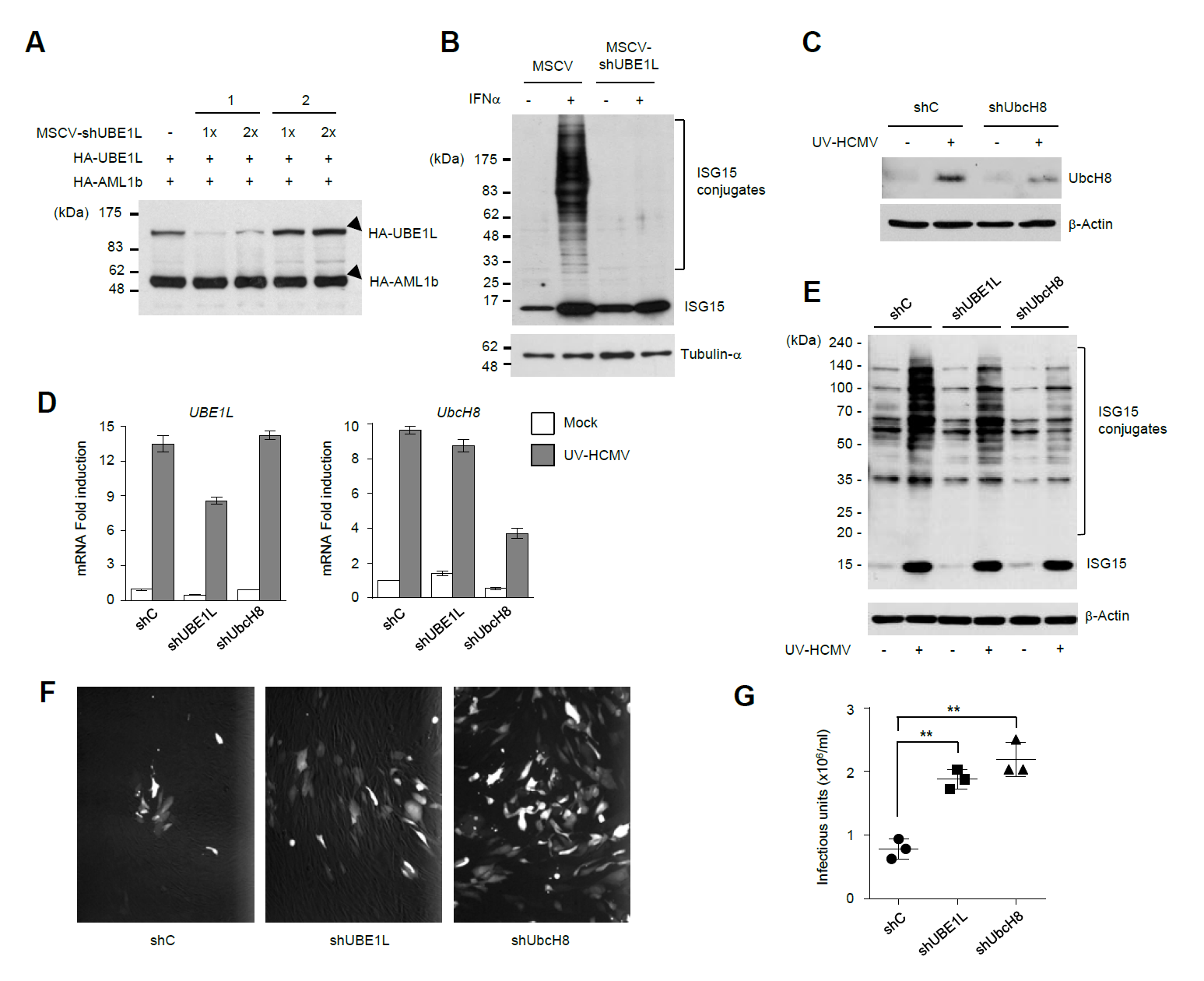

Supplement: S1 Fig — (A) 293T cells were co-transfected with MSCV retroviral vectors expressing shRNA for UBE1L (shUBE1L-1 or shUBE1L-2) and plasmids encoding HA-UBE1L or HA-AML1b as indicated. At 48 h after transfection, cell lysates were immunoblotted with anti-HA antibody. The results showed that shUBE1L-1 specifically reduced UBE1L expression. (B) HeLa cells transduced by MSCV or shUBE1L-1 (hereafter shUBE1L)-expressing MSCV were treated or not with IFNxα (5,000 U/ml) for 24 h. Cell lysates were immunoblotted with antibodies for ISG15 and tubulin-a. (C) Control HF cells or cells expressing UbcH8-specific shRNA were mock-infected or infected with UV-HCMV. The levels of UbcH8 were determined by immunoblotting with anti-UbcH8 rabbit polyclonal antibodies. (D) Control HF cells or cells expressing UBE1L- or UbcH8-specific shRNA were mock-infected or infected with UV-HCMV. The levels of UBE1L and UbcH8 transcripts were determined at 24 h after infection by qRT-PCR. The β-actin transcript levels were used for normalization. Values are the averages of duplicated assays; error ranges are indicated. (E) HF-shRNA cells were infected or not with UV-HCMV at an MOI of 3. At 24 h after infection, immunoblotting was performed with antibodies for ISG15 and β-actin. (F) HF-shRNA cells were infected with the recombinant virus containing the GFP expression cassette (HCMV-GFP) at an MOI of 0.1. GFP images of cells were taken at 7 days after infection. (G) HF-shRNA cells were infected with HCMV at an MOI of 0.1. At 9 days after infection, the viral supernatants were collected and the levels of progeny virions were measured by infectious center assays. Statistical significances were determined using the Student’s t-test and are indicated by **P<0.01. (TIF) [file ppat.1005850.s001.tif]

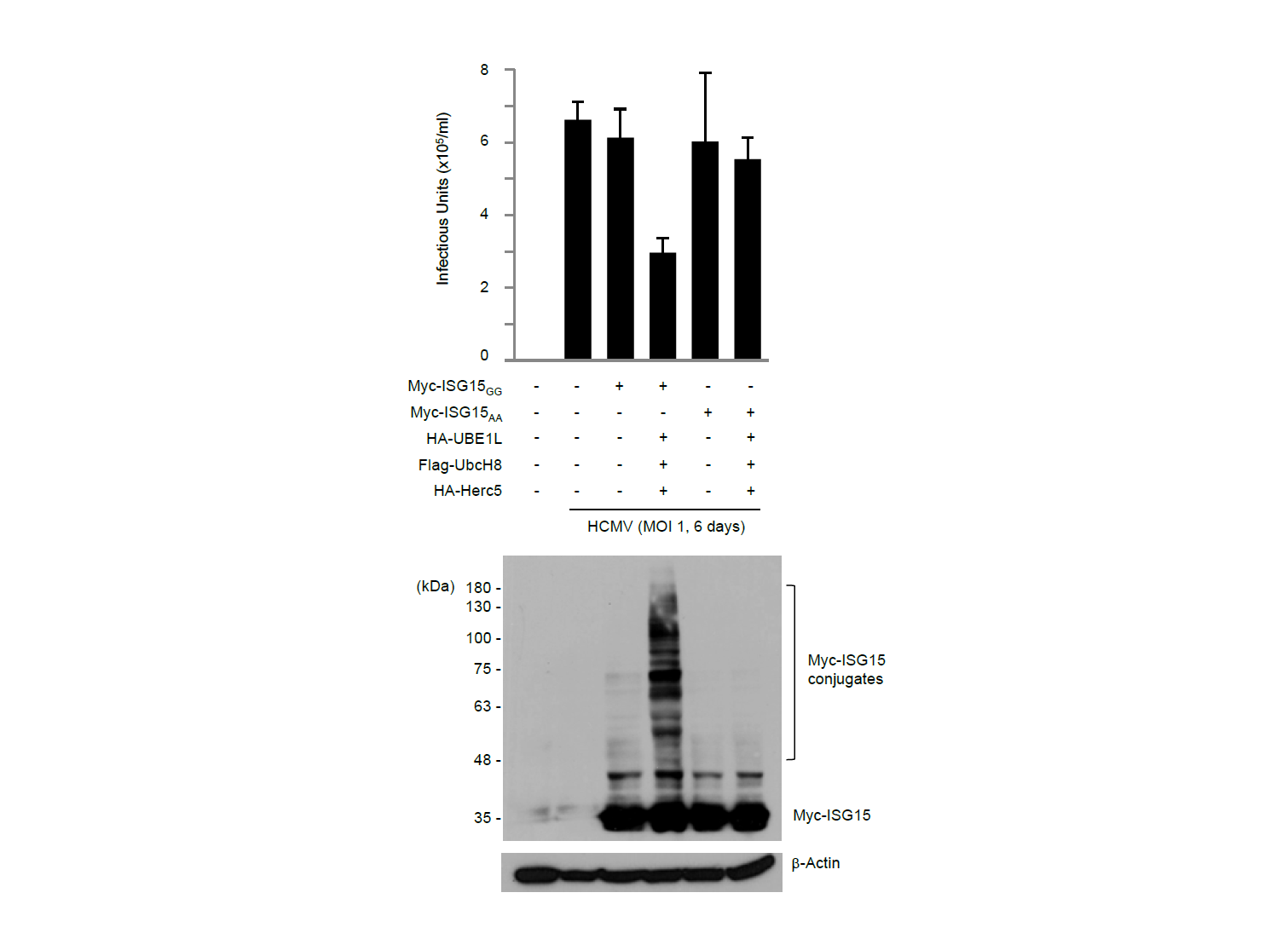

Supplement: S2 Fig — HF cells (2 × 10^5^) were transfected by electroporation with plasmids expressing myc-ISG15GG (or myc-ISG15AA), HA-UBE1L, flag-UbcH8, and HA-Herc5 in combinations as indicated. At 24 h after electroporation, cells were infected with HCMV at an MOI of 1. At 6 days after infection, viral titers in the culture supernatants were measured by infectious center assays (top). The results were shown as averages in two experiments. The levels of exogenously expressed myc-ISG15 proteins, myc-ISG15 conjugates, and β-actin were shown by immunoblotting (bottom). (TIF) [file ppat.1005850.s002.tif]

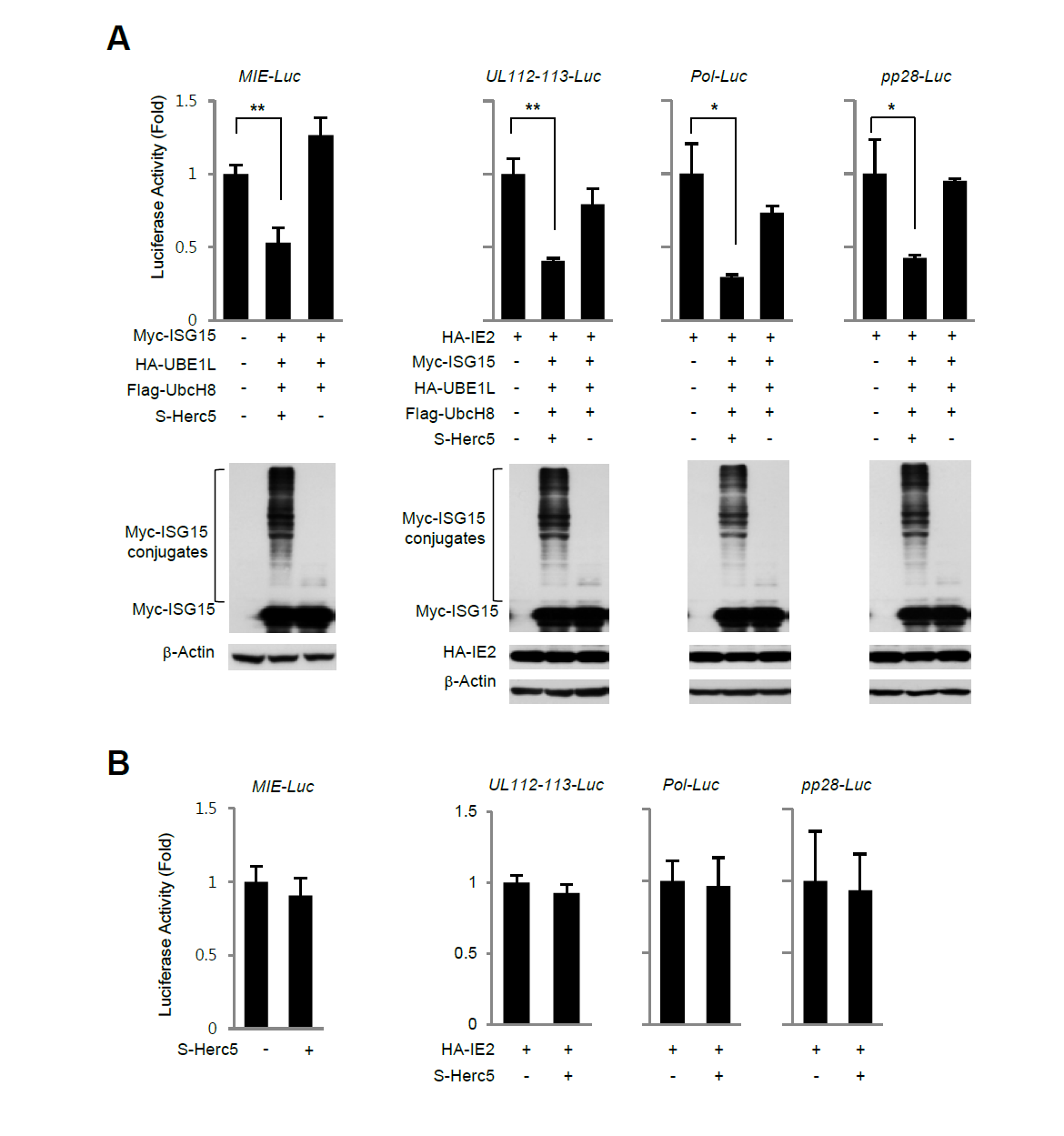

Supplement: S3 Fig — (A-B) HF cells were co-transfected with reporter plasmids containing MIE-Luc, UL112-113-Luc, Pol-Luc, or pp28-Luc reporter gene and effector plasmids as indicated. At 48 after transfection, cell lysates were prepared and assayed for the luciferase activity. The results shown are the mean values for the three independent experiments with standard errors. The expression levels of myc-ISG15 and myc-ISG15 conjugates, HA-IE2, and β-actin in transfected cells were shown by immunoblotting. Statistical significances were determined using the Student’s t-test and are indicated by *P<0.05 or **P<0.01. (TIF) [file ppat.1005850.s003.tif]

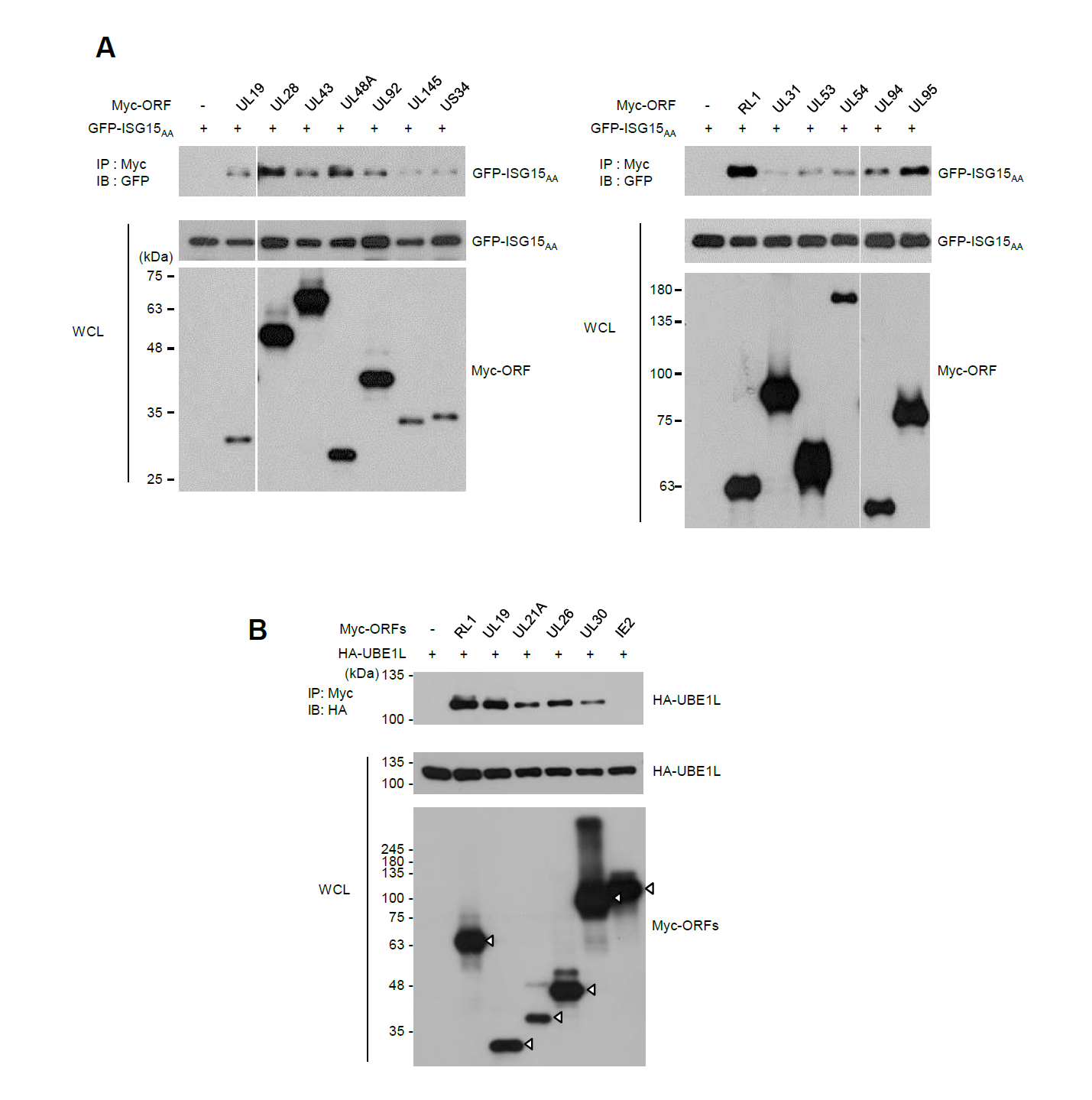

Supplement: S4 Fig — (A) 293T cells were co-transfected with plasmids encoding GFP-ISG15AA or myc-ORFs, as indicated. At 48 h after transfection, cell lysates were prepared and immunoprecipitated with anti-myc antibody, followed by immunoblotting with anti-GFP antibody. To determine the expression levels of each protein, whole cell lysate were also immunoblotted. (B) 293T cells were co-transfected with plasmids encoding HA-UBE1L or myc-ORFs, as indicated. At 48 h after transfection, cell lysates were prepared and immunoprecipitated with anti-myc antibody, followed by immunoblotting with anti-HA antibody. (TIF) [file ppat.1005850.s004.tif]

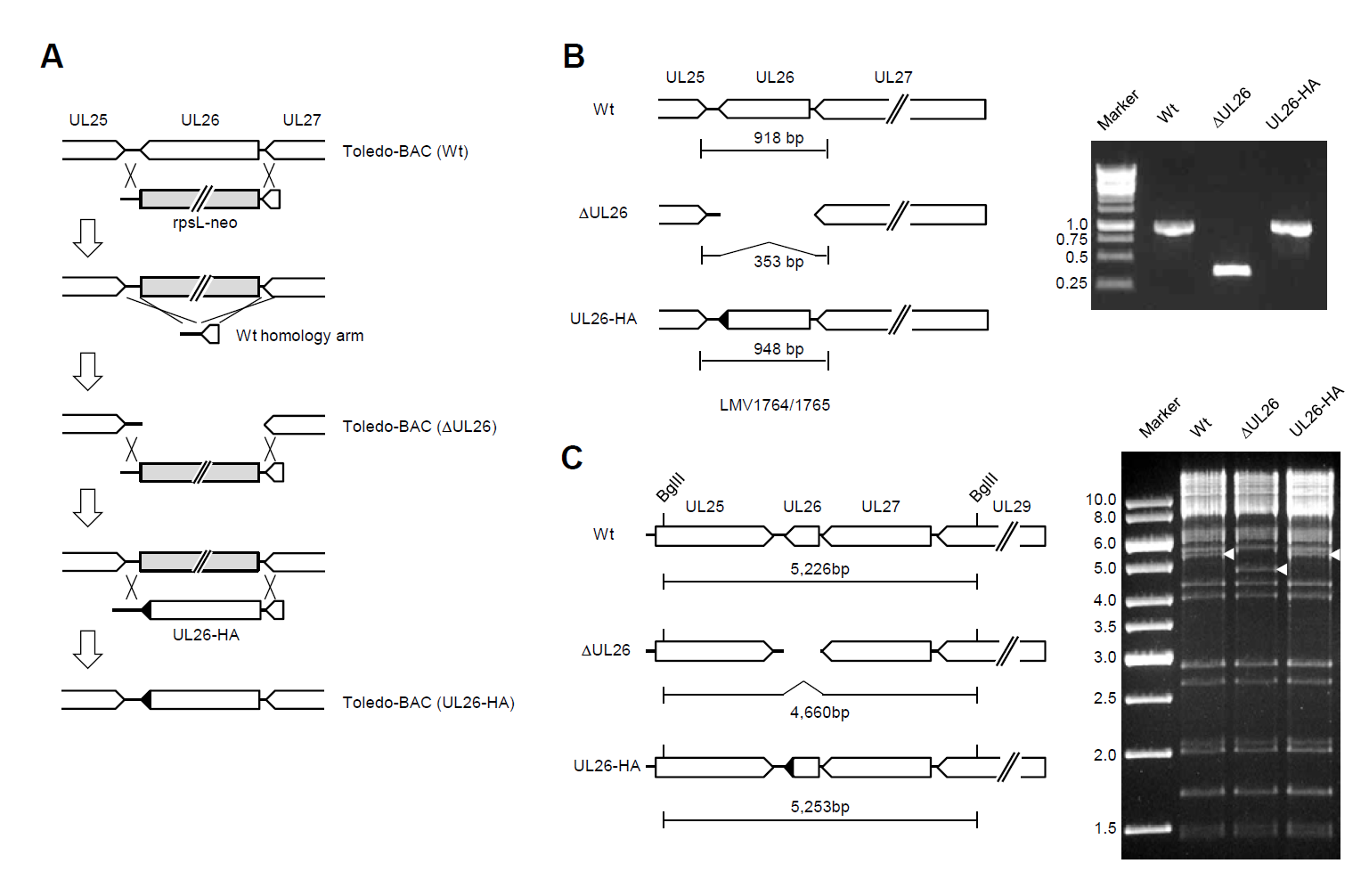

Supplement: S5 Fig — (A) The scheme for Toledo-bacmid mutagenesis. The HCMV (Toledo) bacterial artificial chromosome (BAC) clone (Toledo-BAC)[93] was a gift from Hua Zhu (UMDNJ-New Jersey Medical School, Newark, NJ, USA). The rpsL-neo cassettes were PCR-amplified using LMV1766/1767 primers containing homology arms consisting of 50 nucleotides upstream and downstream of the target region plus 24 nucleotides homologous to the rpsL-neo cassette. The amplified rpsL-neo fragments with homology arms were purified and introduced into E. coli GS243 containing wild-type Toledo-BAC for recombination by electroporation using a Gene Pulser II (Bio-Rad). The intermediate Toledo-BAC constructs containing the rpsL-neo cassette were selected on Luria Broth (LB) plates containing kanamycin. Next, the rpsL-neo cassette was replaced by annealed oligo DNAs (LMV1768/1769) consisting of only homology arms (50 nucleotides upstream and downstream of the target region). The ΔUL26 Toledo-BAC was selected on LB plates containing streptomycin. The mutated regions were amplified by PCR and sequenced to verify the desired mutation. The Toledo-BAC encoding UL26-HA was generated from the mutant Toledo-BAC. First, the rps-neo cassettes flanked by homology arms were inserted again into the mutant Toledo-BAC. Next, DNA fragments containing the wild-type UL26 gene with a HA tag at its C-terminus were PCR amplified by 2-steps using LMV1805/1812 and LMV1805/1772. The amplified UL26-HA gene was then inserted into the Toledo-BAC containing the rps-neo cassette by homologous recombination. The LMV primers used for mutagenesis are listed in S1 Table. (B) The regions containing the UL26 ORF from Wt, ΔUL26, and UL26-HA bacmid DNAs were PCR amplified with LMV1764/1765. (C) Wt, ΔUL26, and UL26-HA bacmid DNAs were digested with BglII and the digestion patterns were compared via agarose gel electrophoresis. The bands corresponding to 5,226 and 5,253 bp from wild-type and UL26-HA bacmids, respectively, and a band of 4,660 bp from ΔUL2 [file ppat.1005850.s005.tif]

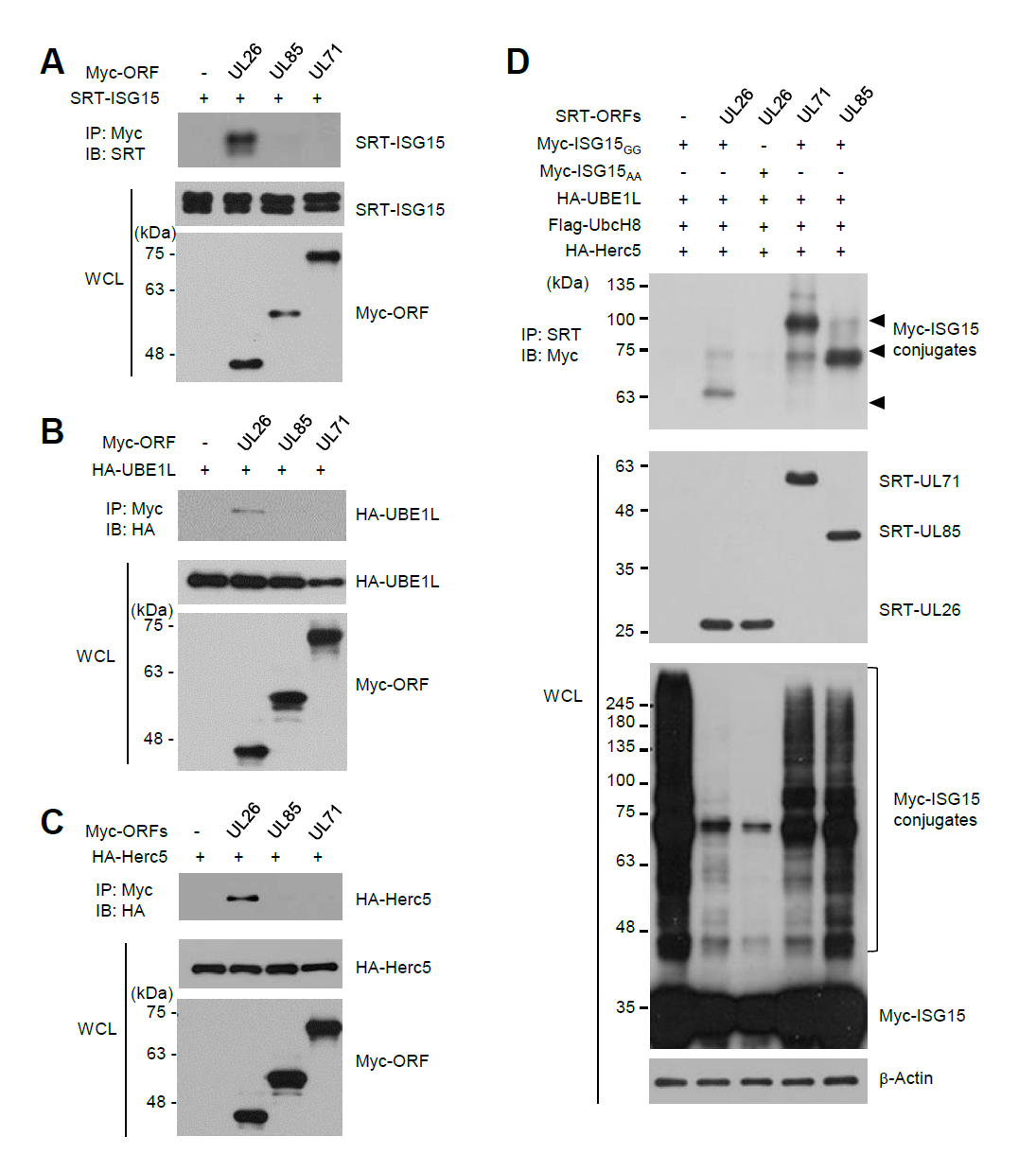

Supplement: S6 Fig — (A-C) 293T cells were co-transfected with plasmids encoding SRT-ISG15, HA-UBE1L, HA-Herc5, or myc-ORFs, as indicated. At 48 h after transfection, cell lysates were prepared and immunoprecipitated with anti-myc antibody, followed by immunoblotting with anti-SRT antibody (A) or anti-HA antibody (B and C). To determine the expression levels of each protein, whole cell lysate were also immunoblotted. (D) Co-transfection/ISGylation assays. 293T cells were co-transfected with plasmid expressing SRT-tagged ORF (UL26, UL85, and UL71), myc-ISG15 (with GG or AA terminus), HA-UBE1L, Flag-UbcH8, or HA-Herc5 as indicated. At 48 h after transfection, cell lysates were immunoprecipitated with anti-SRT antibody, followed by immunoblotting with anti-myc antibody. Whole cell lysates were immunoblotted with anti-SRT antibody to determine the expression levels of each protein. (TIF) [file ppat.1005850.s006.tif]

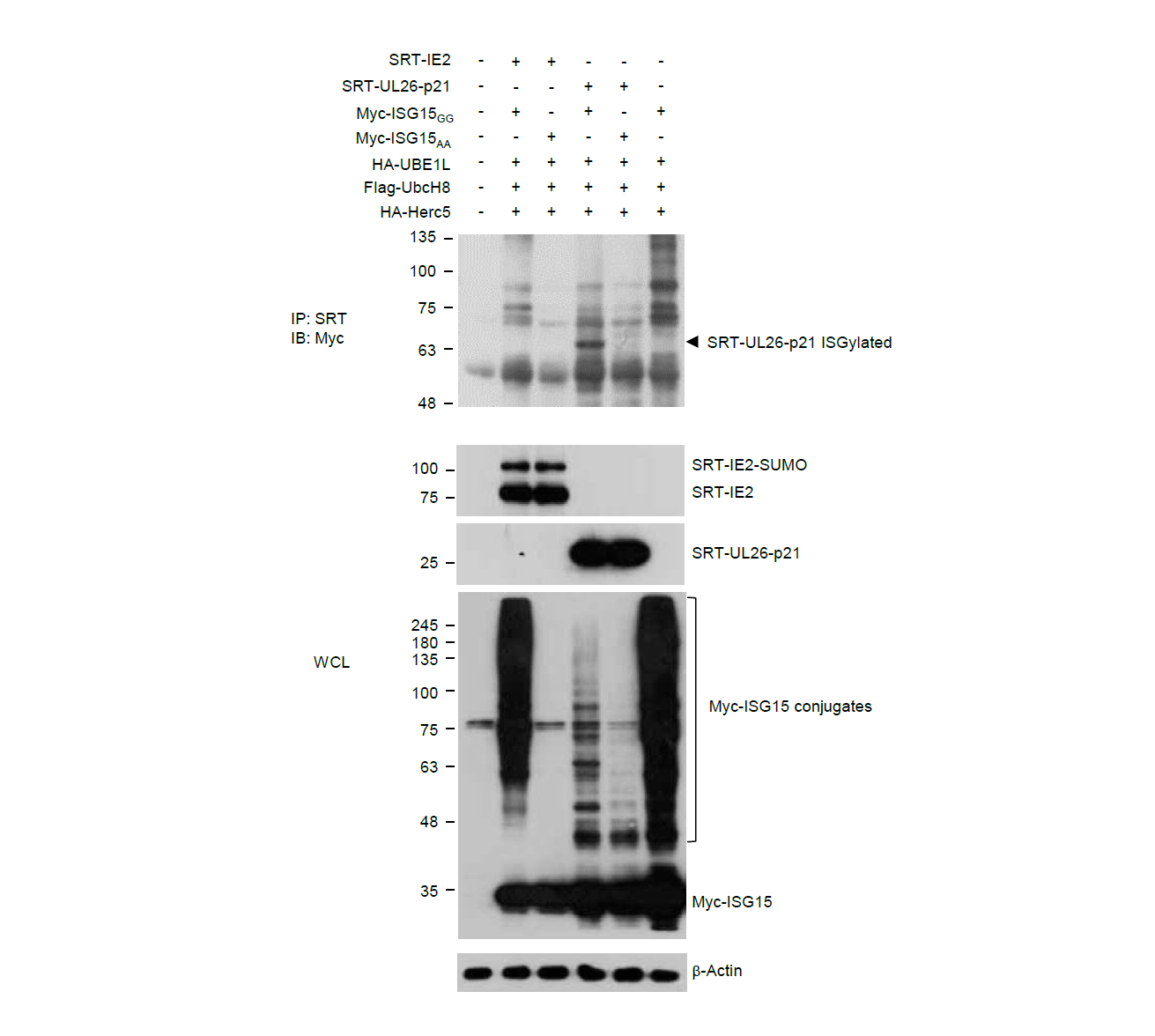

Supplement: S7 Fig — Comparative co-transfection/ISGylation assays for UL26 and IE2 were performed in 293T cells with or without increasing amounts of plasmids expressing SRT-UL26-p21 or SRT-IE2 IE1 as in Fig 2D. Cell lysates were prepared and immunoprecipitated with anti-SRT antibody, followed by immunoblotting with anti-myc antibody. Whole cell lysates were immunoblotted with anti-SRT antibody to determine the expression levels of UL26-p21 and IE2, or with anti-myc antibody to determine the effect of UL26-p21 or IE2 expression on ISGylation. (TIF) [file ppat.1005850.s007.tif]

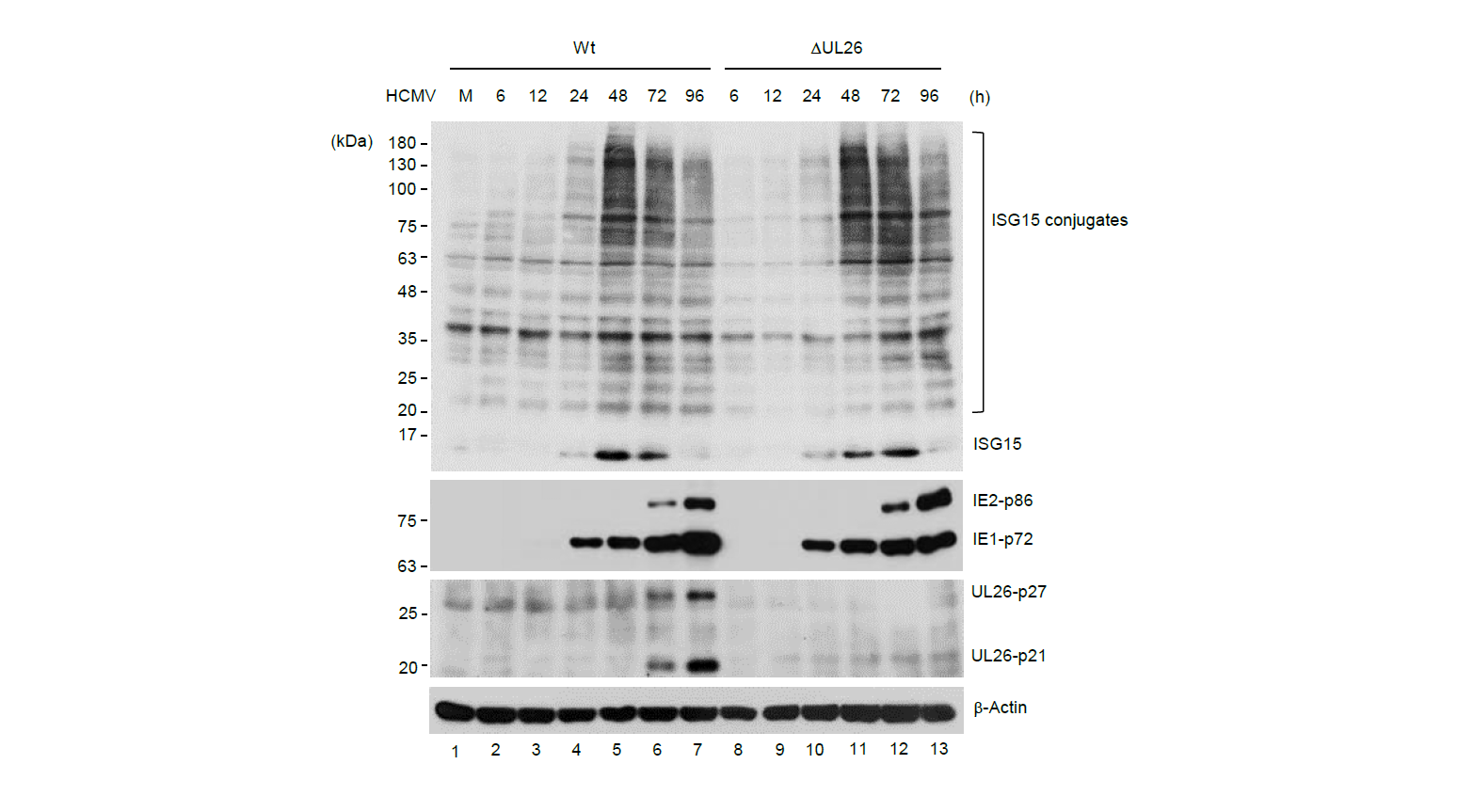

Supplement: S8 Fig — HF cells were mock-infected or infected with wild-type or ΔUL26 mutant virus (Ad169) at an MOI of 0.2. Cell lysates were immunoblotted at the indicated time points with antibodies for ISG15, viral proteins (IE1, IE2, and UL26), and β-actin. (TIF) [file ppat.1005850.s008.tif]

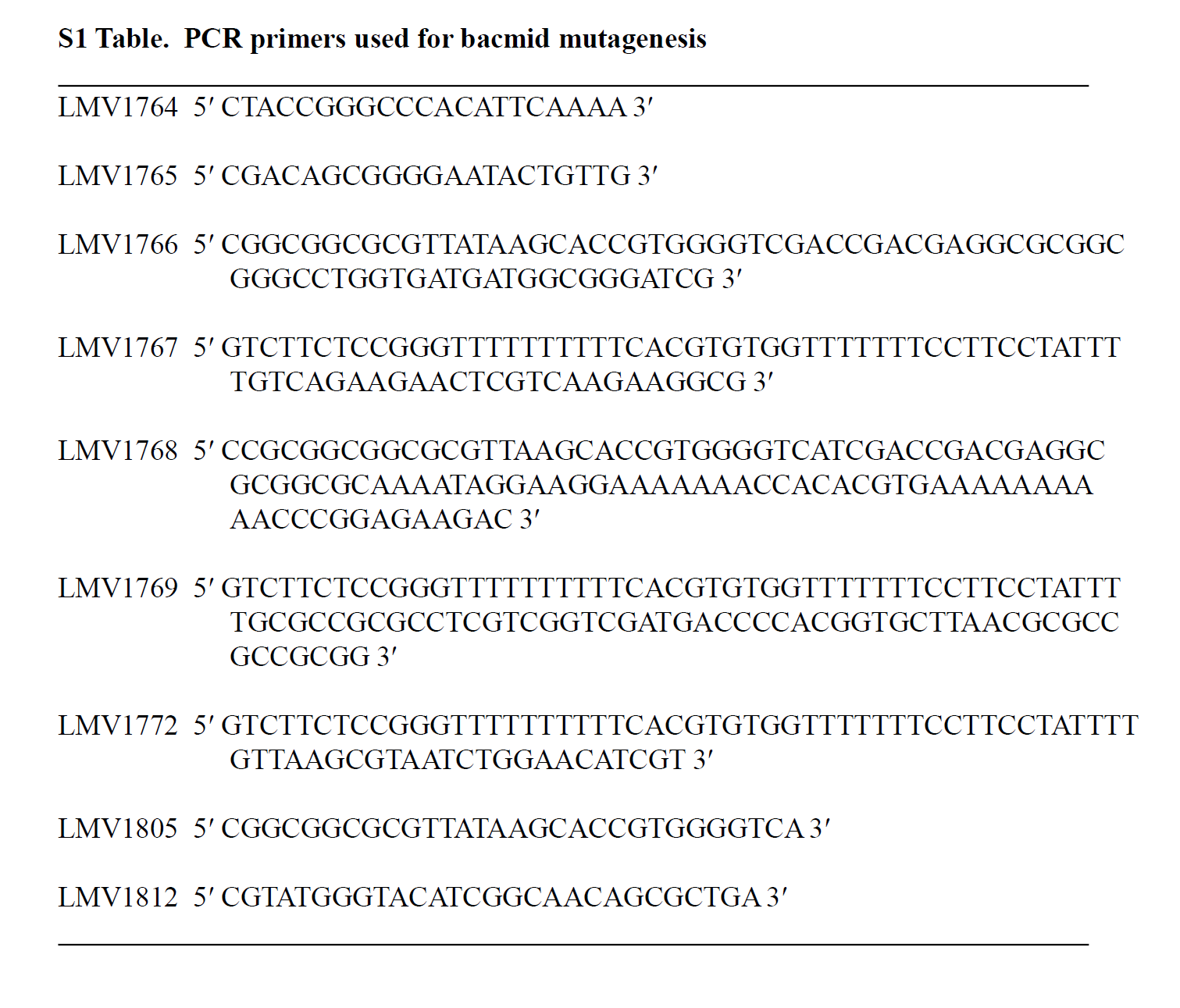

Supplement: S1 Table — (TIF) [file ppat.1005850.s009.tif]

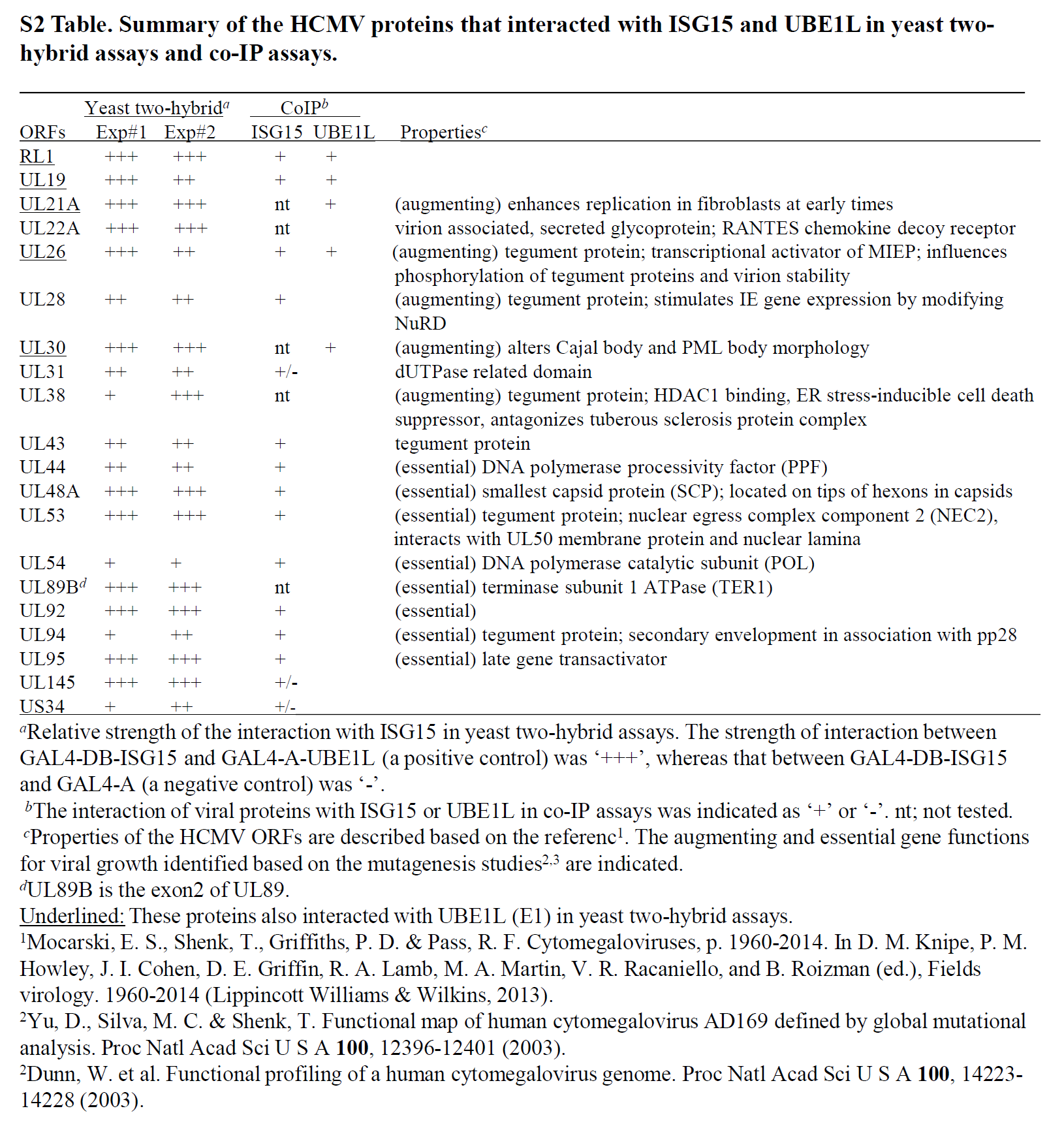

Supplement: S2 Table — (TIF) [file ppat.1005850.s010.tif]
